# The cost-effectiveness of *Wolbachia-*based biocontrol interventions for dengue: A scoping review of the available evidence

**DOI:** 10.1371/journal.pntd.0014395

**Published:** 2026-06-01

**Authors:** Hugo C. Turner, Trinh Manh Hung, Oliver J. Brady, Raman Velayudhan, Ilaria Dorigatti, Hannah E. Clapham

**Affiliations:** 1 MRC Centre for Global Infectious Disease Analysis, School of Public Health, Imperial College London, London, United Kingdom; 2 School of Public Health, Faculty of Health, Medicine and Behavioural Science, The University of Queensland, Brisbane, Australia; 3 Department of Infectious Disease Epidemiology and Dynamics, Faculty of Epidemiology and Population Health, London School of Hygiene and Tropical Medicine, London, United Kingdom; 4 Centre for Mathematical Modelling of Infectious Diseases, London School of Hygiene and Tropical Medicine, United Kingdom; 5 Department of Control of Neglected Tropical Diseases, World Health Organization, Geneva, Switzerland; 6 Saw Swee Hock School of Public Health, National University of Singapore and National University Health System, Singapore, Singapore; QIMR: QIMR Berghofer Medical Research Institute, AUSTRALIA

## Abstract

**Background:**

Dengue incidence has increased sharply worldwide, placing nearly half of the global population at risk. In response, various innovative technologies and interventions, including biocontrol strategies that deploy *Wolbachia*-infected mosquitoes, are being explored. These can be used to either replace the existing mosquito population with one that is less likely to transmit infection or to suppress the existing mosquito population. We conducted a scoping review of economic evaluations of *Wolbachia-*based interventions for dengue control, aimed at summarising assumptions and results of existing studies.

**Methodology/Principal Findings:**

A scoping review of the published literature was conducted on the 29^th^ of April 2024 using the MEDLINE (via OVID), Embase Classic+Embase (via OVID), Global Health - OVID, PubMed, and Econ Lit electronic databases. No date or language restrictions were applied to the searches. We identified nine studies that reported the results of economic evaluations of *Wolbachia-*based interventions for dengue control. The majority (eight out of nine studies) investigated *Wolbachia* replacement-based programmes. Overall, the results were supportive for the use of replacement-based programmes in large urban settings, with the intervention likely to generate cost savings from a societal perspective.

**Conclusions/Significance.:**

The available economic evaluations consistently suggest that *Wolbachia*-based replacement interventions can be cost-effective for dengue control when targeted to densely populated urban areas, and several studies indicate that they can generate substantial long‑term cost savings from a societal perspective. Further research is needed to understand how heterogeneity in epidemiological effectiveness influences long-term projected cost‑effectiveness and to investigate the combination of *Wolbachia-*based interventions with other dengue control/prevention measures (such as vaccination). To support more robust and comparable analyses, we provide recommendations for future studies in this area, emphasising the importance of reporting results disaggregated by cost and outcome components, and making important underlying assumptions related to the intervention more explicit.

## Author summary

Dengue poses a growing global health threat, with nearly half the world’s population at risk. Among emerging control strategies, *Wolbachia-*infected mosquitoes offer a promising biocontrol approach—either by replacing local mosquito populations with less infectious ones or by suppressing mosquito numbers. We conducted the first scoping review of economic evaluations of *Wolbachia-*based interventions for dengue control, identifying nine studies published up to 29^th^ of April 2024. Most focused on replacement strategies, particularly in large urban settings, and found these interventions to be cost-effective from a societal perspective. Our review highlights that the available economic evaluations consistently suggest that *Wolbachia*‑based replacement programmes are highly cost‑effective in high‑burden areas, where the intervention has the potential to generate substantial long‑term cost savings from a societal perspective in many settings. We also identify key drivers of variation across studies and provide recommendations to guide future economic evaluations in this field, emphasising the importance of reporting results in a disaggregated manner and making all underlying assumptions explicit.

## Introduction

Dengue is a mosquito-borne arboviral pathogen that is widespread in tropical and subtropical climates worldwide, mostly in urban and semi-urban areas. The global incidence of dengue has grown dramatically, with approximately half of the world’s population now at risk [1].

Currently, there is no specific therapeutic treatment for dengue; therefore, preventive interventions are vital. Currently, these strategies are predominantly reliant on vector control and increasingly vaccination. However, standard vector control interventions have generally been unable to sustainably control dengue [2], and a range of novel technologies and interventions are being developed. This includes biocontrol strategies involving the release of *Wolbachia-*infected mosquitoes. These can either be used to replace the existing mosquito population with one less likely to transmit infection, generating long-term reductions in transmission (population replacement-based strategy), or suppressing the existing mosquito population by releasing males only (population suppression-based strategy).

To date, *Wolbachia* replacement*-*based programmes have only been conducted in specific mid-sized cities or specific districts within 16 countries. The World Mosquito Program has partnered with governments and communities to deploy *Wolbachia* mosquitoes in 15 countries since 2011, leading to the successful establishment of the *wMel Wolbachia* strain in local *Aedes aegypti* populations, covering over 11 million people (as of March 2024) [3]. There is also a separate *Wolbachia* replacement programme in Malaysia using the *wAlbB Wolbachia* strain [4]. Currently, three countries (China, Singapore, and the United States [5–7]) are using *a Wolbachia* suppression-based strategy, likely because of the perceived greater compatibility of a suppression-based strategy with their existing intensive and long-term efforts to suppress mosquito populations [8].

Countries are reaching the stage of considering the large-scale use of these types of interventions in a more programmatic context. Therefore, it is vital to understand the current evidence base regarding the cost-effectiveness of these interventions and the current research gaps.

This paper aims to perform an in-depth review of the economic evaluations that have been conducted on *Wolbachia-*based interventions to control dengue. This will inform the evidence base for adopting these interventions and highlight important areas that require further investigation and recommendations for future studies.

## Method

We conducted a scoping review of the health economic evaluations of the use of *Wolbachia*-based interventions for dengue control. The aim was to identify and synthesise the results of studies in this area, as well as to determine possible gaps requiring further research. Because this scoping review was undertaken as an iterative exploratory exercise intended to map the emerging evidence, no protocol was registered at the time the review was initiated.

### Search strategy and selection criteria

Publications were collected by searching the MEDLINE (via OVID), Embase Classic+Embase (via OVID), Global Health - OVID, PubMed, and Econ Lit databases on the 29^th^ of April 2024. The search terms included variants of the following keywords (dengue, *Wolbachia*, biocontrol, economic evaluation, cost-effectiveness analysis, cost-utility analysis, cost-benefit analysis, cost-consequence analysis, and cost-minimisation analysis) without any date- or language- restrictions. Additional searches of the grey literature were performed as outlined in the S1 File.

**Fig 1:**
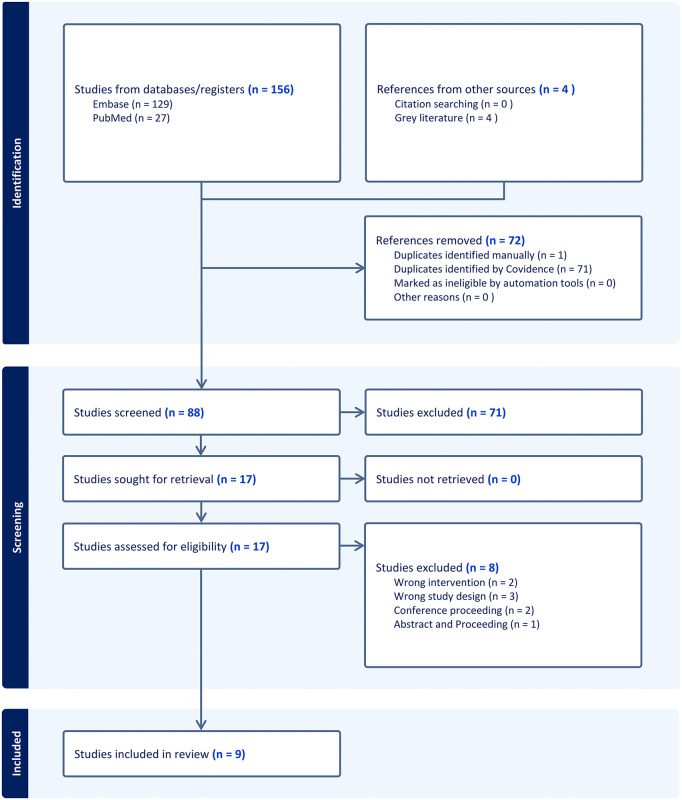
Flow diagram outlining the inclusion and exclusion of the identified studies. *A PRISMA checklist is provided in* S2 File [10].

The retrieved citations were uploaded to Covidence, a web-based systematic review software [9] to identify and remove duplicates. The titles and abstracts of all the articles were scanned to identify relevant studies by two reviewers (HCT and TMH). The bibliographies of related papers were also searched to identify additional articles not initially retrieved from the databases. The full texts of the identified studies were then reviewed for eligibility by two reviewers (HCT and TMH). Any studies with uncertainty regarding their inclusion were discussed and resolved by the reviewers. The full selection process is illustrated in Fig 1. More detailed information on the search terms and PRISMA checklist are provided in the S2 File [10]. As stated in the relevant conflict of interest section, it is important to note that HCT has received research funding from The World Mosquito Program in the past. The World Mosquito Program had no involvement in this paper (other than being a potential source of studies within the search of the grey literature).

Based on the database and grey literature searches (the latter including pre-prints), we included studies that conducted an economic evaluation (i.e., cost-utility analysis, cost-effectiveness analysis, or cost-benefit analysis [11]) related to the use of the *Wolbachia-*based intervention to control dengue (both suppression and replacement-based strategies). Although no language restrictions were applied at the search stage, non‑English full texts were excluded during screening because translation resources were not available. Reviews/systematic reviews, intervention costing studies, and conference abstracts were also excluded.

In cases where a non–peer‑reviewed study was identified, and a peer‑reviewed version was later published, we also considered the peer‑reviewed version when extracting data, even if it was published after the search timeframe.

### Data extraction and output

Methodological and contextual data from each study were systematically extracted by two independent reviewers (HCT and TMH). Key methodological details included the study setting, base case time horizon, the modelling approach used to estimate effectiveness, the primary outcome measure, and the discount rate applied in the base case results were extracted. Additional relevant assumptions were documented, such as the baseline burden of symptomatic cases in the absence of intervention, the effectiveness of the intervention, the size of the release area, the population covered, the cost of the intervention and the corresponding cost year (S3 File).

We grouped the scale of the intervention into three categories: National, large scale (targeting at least 200,000 people), and small scale (targeting under 200,000 people).

The results of the studies were summarised by extracting their reported cost-effectiveness ratios and benefit-cost ratios. The cost-effectiveness ratios were stratified by the different perspectives employed [12]. All of the benefit-cost ratios extracted related to the societal perspective (S1 File: S1 Box).

Cost values (and economic benefits) were adjusted for inflation to 2024 prices using the United States (US) gross domestic product (GDP) deflators [13,14]. Incremental cost-effectiveness ratio (ICER) values were not adjusted and reported with the corresponding cost year.

To provide overarching, high-level conclusions, cost‑effectiveness ratios were compared against a threshold of 0.5 times a country’s per‑capita GDP [15,16] (values taken from the World Bank [17]). This benchmark is gaining traction as an alternative to the earlier (widely criticised) benchmarks that classified interventions as “cost‑effective” if they fell below 1–3 times per‑capita GDP per disability-adjusted life year (DALY) averted [18]. Furthermore, an empirical analysis of historical spending across 174 countries found that 51% implicitly used thresholds of cost per quality-adjusted life year (QALY) gained of 0.5 times their per capita GDP [19]. However, it remains important to recognise that appropriate cost-effectiveness thresholds continue to be debated, and GDP-based benchmarks have well-recognised limitations that should be considered when interpreting these findings [20–23].

### Quality assessment

Quality assessment of the identified relevant studies was undertaken using the CHEQUE tool [24]. This was conducted by two independent reviewers (TMH and HCT) following the procedures outlined for screening and data extraction.

## Results

We identified 160 potentially relevant studies (Fig 1). After removing duplicate papers in Covidence, a total of 88 studies remained. After title and abstract screening, 71 papers were excluded. The remaining 17 studies underwent full-text screening, and after the further exclusion of eight papers, nine relevant studies were included in this scoping review. A summary of the PRISMA chart is shown in Fig 1.

A summary of the key features of the identified studies is presented in Table 1. The majority of the studies were cost-utility analyses, with several also presenting benefit-cost ratios as an additional output. Some studies presented both cost-utility and cost-benefit analyses [25–27]. In terms of intervention strategy, eight were related to replacement [25–33], and only one was related to suppression [34]. Two of the studies [32,33] considered the potential combination of *Wolbachia*-based interventions with dengue vaccination programmes.

**Table 1:** Summary of the key features of the identified studies.

Study	Settings	Strategy investigated	Study type	Base case time horizon	Modelling approach to estimate effectiveness	Outcome measure and weight source	Base case discount rate	Consider cost savings related to the reduced need for existing dengue-related vector control
Soh *et al.* 2021	Singapore	*Wolbachia* suppression-based programme	CUA	2010-2020 (11 years)	Static model: Retrospective projection	DALYs averted – disability weights based on the Meltzer *et al.* approach [45]	3%	No
Brady *et al.* 2020	Indonesia (Yogyakarta City, Yogyakarta Special Autonomous Region, Jakarta, and Bali)	*Wolbachia* replacement-based programme	CUA – with BCR reported	10-year post-release	Dynamic model: Population-based model	DALYs averted – disability weights from Zeng *et al*. [44]	3%	No
Turner *et al.* 2023	Vietnam (ten high burden cities)	*Wolbachia* replacement-based programme	CUA – with BCR reported	20-year post-release	Static model: Population-based projection	DALYs averted – disability weights from Zeng *et al*. [44]	3%	Yes
Shepard *et al*. 2020	Fiji (Suva) and Vanuatu (Port Vila)	*Wolbachia* replacement-based programme	CBA & CUA	10 years	Static model: Population-based projection	DALYs averted – unclear	3%	No
Barbosa *et al*. 2023	Brazil (State of Goiás)	*Wolbachia* replacement-based programme	CUA	13 years	Static model: Decision tree model	QALY gained - utility weights based on Suwantika *et al*. [46]	5%	Yes
Zimmermann *et al*. 2024	Brazil (Seven priority cities)	*Wolbachia* replacement-based programme	CUA – with BCR reported	20 years	Static model: Microsimulation markov model	DALYs averted – disability weights from GBD (post 2013 approach)	5% for costs, health benefits not discounted	Yes
Shepard *et al.* 2024 (and Shepard *et al.* 2025)	Colombia (11 priority cities)	*Wolbachia* replacement-based programme	CBA & CUA	10 years	Static model: Population-based projection	DALYs averted – disability weights from Zeng *et al*. [44]	3%	Yes
Knerer *et al.* 2020	Thailand	A combination of vaccination and a *Wolbachia*-based replacement programme	CUA	10 years	Dynamic model: Population-based model	DALYs averted – disability weights 0.211 and 0.5 for symptomatic cases of DF and DHF/DSS, respectively	3%	No
Suwantika *et al.* 2020	Indonesia	A combination of vaccination and a *Wolbachia*-based replacement programme	CUA	10 years	Static model: Age-structured decision tree model simulating a cohort of children	QALY gained - utility weights based on the disability weights estimated by Zeng *et al*. [44]	3%	No

*BCR: Benefit–Cost Ratio, CBA: Cost–Benefit Analysis, CUA: Cost–Utility Analysis, DALY: Disability-Adjusted Life Year, DF: Dengue Fever, DHF/DSS: Dengue Haemorrhagic Fever / Dengue Shock Syndrome, GBD: Global Burden of Disease, QALY: Quality-Adjusted Life Year.*

### Baseline disease burden

Due to epidemiological and entomological differences, it is expected that the baseline disease burden of dengue varies from area to area. It should also be noted that symptomatic dengue cases are underreported and that the proportion of symptomatic infections reported to surveillance varies between and within countries; therefore, adjustments and modelling approaches are needed when evaluating the population-level impact of dengue interventions. The corresponding actual disease burden estimated will vary depending on the approach employed.

As expected, the assumed baseline incidence of infection varied between the settings investigated (Table 2). However, in some cases, this was not clearly reported. The approach used to approximate the baseline infection burden and adjust for underreporting was also variable (Table 2). However, it is encouraging that all the studies adjusted for underreporting in some form rather than using reported case numbers directly. Several studies adjusted the reported number of cases with expansion factors (the ratio of the estimated true number of symptomatic dengue cases to the reported number of dengue cases). Brady *et al*. [28] used an unweighted ensemble of multiple previous approaches to obtain consensus estimates of the burden of dengue in Indonesia (taken from O’Reilly *et al*. [35]). In Turner *et al.* [29], the baseline burden was based on the incidence of dengue estimated by the Global Burden of Disease (GBD) 2019 study [36] for Vietnam, which was distributed sub-nationally based on the mapping projections by Bhatt *et al*. [37]. Shepard *et al*. [26] used burden estimates derived from projections by the GBD study.

**Table 2:** Summary of the assumed baseline burden and the base case effectiveness and cost of the *Wolbachia* intervention.

Study	Setting	Baseline burden of symptomatic cases in the absence of the intervention	Base case – symptomatic cases per 100,000 per year	Base case effectiveness (percentage reduction in dengue cases)^1^	Base case duration of effectiveness assumed for base case results	Release area (Km^2^) covered	Population covered - millions	Scale	Base case intervention cost per person (US$ 2024)	Base case total intervention cost - US$ millions (2024 prices)	Original cost year
Soh *et al.* 2021	Singapore	Varied year by year between 2,767–35,315 cases	–	40-80%	Not applicable	Not directly reported	Not directly reported: inferred to be 5,076,732 based on the population of Singapore in 2010 [13]	Large	Not directly reported: estimated to be approximately US$59^4^	Not directly reported: estimated to be 297.34 over 11 years^5^	2010
Brady *et al*. 2020^2^	Yogyakarta City, Indonesia	14,488 per year	3,150	Modelled: 94.4% (within treated areas)	10 years*	37.24	0.46	Large	Accel: 15.41	Accel: 7.09	2018
									Seq: Not applicable	Seq: Not applicable	2018
	Yogyakarta Special Autonomous Region, Indonesia	93,604 per year	2,890	Modelled: 87.2% (within treated areas)	10 years*	909	2.14	Large	Accel: 17.43	Accel: 37.23	2018
									Seq: 15.57	Seq: 33.26	2018
	Jakarta, Indonesia	444,528 per year	3,970	Modelled: 65.7% (within treated areas)	10 years*	764	11.19	Large	Accel: 14.47	Accel: 161.74	2018
									Seq: 9.04	Seq: 101.11	2018
	Bali, Indonesia	117,840 per year	2,890	Modelled: 82.8% (within treated areas)	10 years*	964	2.44	Large	Accel: 25.74	Accel: 62.68	2018
									Seq: 17.38	Seq: 42.32	2018
Turner *et al.* 2023	Vietnam (ten high burden cities)	363,086 per year	1,814	75%	20 years*	1,369	20.02	Large	10.16	203.38	2020
Shepard *et al.* 2020	Suva, Fiji	2,500 per year	1,111	75%	10 years*	Not directly reported	0.225	Large	14.68	3.30	2018
	Port Vila, Vanuatu	605 per year	1,120	75%	10 years*	Not directly reported	0.054	Small	53.99	2.91	2018
Barbosa *et al.* 2023	Brazil (State of Goiás)	1.99 million cases over 13 years (an average of 153,343 per year)	–	Not clearly stated (75% based on the reported results)	10 years	2,864	7.3	Large	7.10	57.44	Unclear (assumed to be 2022)
Zimmermann *et al*. 2024	Brazil (Seven priority cities)	1.76 million dengue cases over the 20-year time horizon (an average of 88,134 per year)^3^	–	77.10%	5, 10, and 20 years	2,801	22.12	Large	2.62	Not directly reported	2022
Shepard *et al*. 2024 (and Shepard *et al.* 2025)	Colombia (11 priority cities)	94,239 per year	1,071	37.5% 1 year since deployment, 75% ≥ 2 years since deployment	10 years*	412	8.34	Large	5.62	46.86	2020
Knerer *et al.* 2020	Thailand	7.2 million cases over 10 years (an average of 714,700 per year)	–	Modelled: Value not stated	10 years*	Not stated; assumed to be 513,120	63.5	National	5.81	357.15 (*Wolbachia* alone)	2013
Suwantika *et al.* 2020	Indonesia	Unclear	–	86%	10 years	Not directly reported	4.70 (children)	National	3.64	Not directly reported	2018

* The duration of effect was varied in the sensitivity analysis. ^1^ *Values only pertain to the Wolbachia intervention assumptions.*^2^ *Brady et al.* [28] *considered two strategies: the accelerated (denoted as “Accel”) strategy was where every area conducted the intervention simultaneously and independently (total programme length 13 years), vs a “sequenced” (denoted as “Seq”) strategy, where releases were spread over 10 years with certain centralised resources moved or reutilised across different locations.* ^3^ *Excluding inapparent infections (defined as those that were asymptomatic or did not seek formal medical treatment (self-care)).* ^4^ *Based on the assumed population size of Singapore and the estimated total cost of the intervention.* ^5^*Based on the reported yearly value of US$31.24* million *(discounted at 3% per year).*

### Effectiveness of intervention

A summary of the assumed effectiveness of *Wolbachia* deployments is presented in Table 2. The only evaluation of a suppression-based programme assumed a reduction in the incidence of dengue cases varying between 40–80%. This was based on the results of field trials of this intervention in Singapore [38] (Table 2).

Several of the evaluations of the replacement-based strategy assumed the effectiveness of *Wolbachia* deployments in reducing the incidence of symptomatic cases to be 75–77%, based on data from a cluster randomised trial and quasi-experimental studies in Yogyakarta (with the *wMel* strain) [39,40]. Brady *et al*. [28] used data from vector competence studies to model *Wolbachia*-induced changes in force of infection, which could then be projected to areas with different force of infection values and thus produce locally varying estimates of effectiveness. The values varied between 65.7% and 94.4% (within the treated areas).

The duration of the effectiveness of the replacement-based strategy was typically assumed to last 10 years for the base case results. This assumption was often included within the sensitivity analysis. This is a key parameter in the economic analysis of the replacement-based strategy. It should be noted that 10 years was used in the first studies in this area, as that was approximately how long *wMel* had persisted in northern Australia at the time they were conducted. This could therefore be justifiably increased to closer to 15 years now.

### DALY calculations

Seven of the nine studies used DALYs averted as their effectiveness measure (Table 1). DALYs are calculated as the sum of two components: years of healthy life lost due to disability (YLDs) and years of life lost due to premature mortality (YLLs) [41,42]. Within a DALY calculation, YLDs are calculated using a disability weight factor ranging between 0 and 1, which reflects the severity of the disease sequelae, with 0 representing perfect health and 1 representing death. The disability weights used for dengue DALY calculations have changed significantly over time [43]: a summary of the different weight values is provided in S1 File: S2 Box. Interestingly, most of the economic evaluations we identified did not use the disability weights officially designated for dengue in the current GBD studies, which were only used by Zimmermann *et al.* [31]. Four of the studies used the disability weights estimated by Zeng *et al.* [44] (Table 1). Soh *et al.* [34] used a different approach based on what was developed by Meltzer *et al.* [45] (developed before the GBD 2004 update). Knerer *et al.* [32] used the disability weights outlined within the GBD 2004 update.

### QALY calculations

Two studies used QALYs gained as the main outcome. The QALY utility weights within Suwantika *et al.* [33] were derived from the literature, adjusted from the DALYs weights estimated by Zeng *et al*. [44]. The QALY utility weights used by Barbosa *et al*. [30] were based on the utility weights reported by Suwantika *et al*. [46]. These were calculated using a retrospective questionnaire administered to 144 patients in three cities (Jakarta, Bandung, and Yogyakarta), which represent regions with a high prevalence of dengue infection in Indonesia. They estimated QALY losses in outpatients, inpatients, and fatal cases to be 0.00004, 0.00018, and 1 (per year), respectively.

### Cost of the intervention

**Fig 2:**
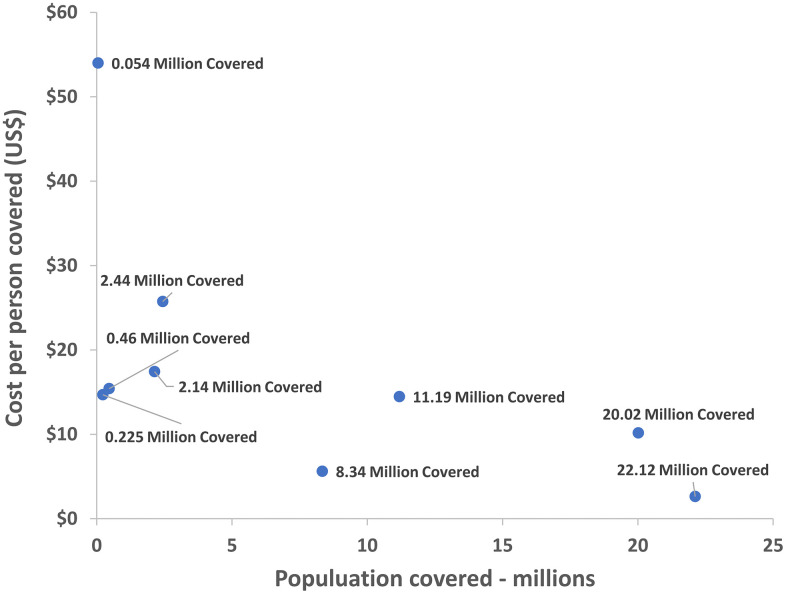
The reported cost per person covered compared to the population covered. *Labels next to each point indicate the population size (millions) for that setting. Only costs related to primary data were considered (hypothetical cost benchmarks/targets were not plotted). Uses the data related to the accelerated scenario for Brady et al.* [28].

A summary of the reported costs and areas covered by the intervention is presented in Table 2 and Fig 2.

The evaluation of the suppression-based programme in Singapore assumed it would cost US$31.2 million per year (2024 prices) [34]. Note that this is an ongoing cost in the future, as the impacts are not self-sustaining. The cost per person covered was not directly reported; however, based on the population of Singapore (5,076,732 in 2010 [13]), it would cost approximately US$59 per person covered (2024 prices) over the 11-year intervention period examined (Table 2).

The assumed base case total cost per person covered within the evaluations of the replacement-based strategy varied between US$2.62-52.99 (2024 prices) per person covered over the course of the programme (Table 2). The majority of the costs were related to the preparation and release phases of the deployment [28,29], occurring within the first two years. A key driver of the cost was the population size/area covered (the highest cost per person was related to covering a small area/population) (Table 2). In the evaluations of larger deployments, the projected cost was often less than US$10 per person covered. In terms of the source of the cost data, some studies were based on budgets/incurred costs [25–29,31,34], whereas others were based on hypothetical cost benchmarks/targets [32,33].

In addition, Brady *et al*. [28] projected that conducting releases over a longer sequenced programme could reduce the total cost by 11–38% (albeit with a delay in benefits).

### Cost-effectiveness/cost-benefit estimates

The status quo (i.e., continuing existing dengue control measures) was used as the comparator. *Wolbachia*-based biocontrol interventions were projected to generate notable economic benefits from averted costs of dengue illness (e.g., averted medical costs and prevented productivity losses). The type of economic benefits (cost savings) considered depended on the perspective (S1 File: S1 Box) [12]. Four studies [25,27,29–31] also considered cost savings related to the reduced need for existing dengue-related vector control measures, while the others assumed that these costs would remain unchanged or did not consider them.

In terms of output, rather than ICERs, some studies presented the results in terms of gross and/or net cost-effectiveness ratios:

1. Gross cost-effectiveness ratios were calculated by dividing the investment cost of the intervention by the number of DALYs averted (the cost is not incremental and therefore no cost savings are accounted).
2. Net cost-effectiveness ratios were calculated with the relevant cost savings being deducted from the investment cost of the intervention before it was divided by the number of DALYs averted. Which cost types are included in the cost savings depends on the perspective of the analysis [12,47] (S1 File: S1 Box). This would be equivalent to a traditional ICER-based output and is denoted as ICERs in the main results table (Table 3).

**Table 3:** Reported cost-effectiveness ratios stratified by the assumed perspective (cost year variable).

Study	Setting	0.5 GDP per capita (2024)	Gross cost-effective ratio (US$)	ICER - health care provider or payer perspective (US$)	ICER - health sector perspective (US$)	ICER – societal perspective (US$)	Societal benefit-cost ratio (US$)	Cost year
Soh *et al.* 2021	Singapore	45,337	40% effectiveness: 100,907 80% effectiveness: 50,453	NA	NA	Not directly reported	Not directly reported; estimated to be 1.27 (at 40% effectiveness) 2.65 (at 80% effectiveness)^1^	2010
Brady *et al.* 2020^1^	Yogyakarta City, Indonesia	2,463	Accel: 1,831	NA	NA	Accel: Cost saving	2.35	2018
			Seq: 1,519			Seq: Cost saving		2018
	Yogyakarta Special Autonomous Region, Indonesia		Accel: 2,133	NA	NA	Accel: Cost saving	1.44	2018
			Seq: 2,168			Seq: Cost saving		2018
	Jakarta, Indonesia		Accel: 1,566	NA	NA	Accel: Cost saving	3.4	2018
			Seq: 1,111			Seq: Cost saving		2018
	Bali, Indonesia		Accel: 2,996	NA	NA	Accel: 671	1.35	2018
			Seq: 2,366			Seq: 64		2018
Turner *et al.* 2023	Vietnam (ten high burden cities)	2,359	1,118	709	420	Cost-saving both when including and excluding the productivity gains related to prevented excess mortality	1.75	2020
Shepard *et al.* 2020	Suva, Fiji	3,213	4,980	NA	N	Not directly reported	1.67	2018
	Port Vila, Vanuatu	1,705	20,193	NA	Not directly reported	Not directly reported	0.19	2018
Barbosa *et al*. 2023	State of Goiás, Brazil	5,155	NA	13,992	NA	Cost saving	NA	Unclear (assumed to be 2022)
Zimmermann *et al*. 2024	Brazil (Seven priority cities)	5155	NA	Overall value not reported – results for individual cities shown in the study’s appendix	Overall value not reported – results for individual cities shown in the study’s appendix	5-year effect: Cost saving	Not directly reported	2022
						10-year effect: Cost saving		2022
						20-year effect: Cost saving		2022
Shepard *et al.* 2024 (and Shepard *et al.* 2025)	Colombia (11 priority cities)	3,960	NA	NA	Cost saving	Cost saving	4.68	2020
Knerer *et al.* 2020	Thailand	3,673	NA	Wol alone: 1,399	NA	Wol alone: 343	NA	2013
						Wol+Vaccination with 40% coverage: 11,462		2013
						Wol+Vaccination with 60% coverage: 503,966		2013
						Wol+Vaccination with 80% coverage: 514,432		2013
Suwantika *et al.* 2020	Indonesia	2,463	NA	Wol+Vaccination: 4,639	Wol+Vaccination: 4,460	NA	NA	2018

ICER: Incremental cost-effectiveness ratios. Wol: Wolbachia. NA: Not applicable. The different perspectives are outlined in S1 File: S1 Box. The corresponding cost years are summarised in Table 2.

^1^Brady et al. [28] considered two strategies: the accelerated (denoted as “Accel”) strategy was where every area conducted the intervention simultaneously and independently (total programme length 13 years), vs a “sequenced” (denoted as “Seq”) strategy, where releases were spread over 10 years with certain centralised resources moved or reutilised across different locations.

^1^The BCR were estimated based on the projected total cost (Table 2) and the sum of the reported yearly costs averted (S1 File: S1 Table). Both were discounted at 3% per year.

The cost-effectiveness ratios stratified by the different perspectives employed are summarised in Table 3 (with the corresponding economic benefits reported in S1 File: S1 Table). For this intervention, the broader the perspective, the lower the cost-effectiveness ratio. This is because the broader the perspective, the higher the averted cost of illness associated with dengue cases; with the societal perspective including the estimated monetary value of the prevented productivity losses that would have been associated with a dengue case. For the same reason, the projections based on gross cost‑effectiveness ratios (ignoring all cost offsets) appeared less favourable than ICER-based output. Differences in the chosen analytic perspective as well as the types of ratios reported, made further direct comparison across studies challenging. As a result, further quantitative synthesis of the cost‑effectiveness ratios—and any meaningful graphical presentation of these findings—was not feasible.

**Fig 3:**
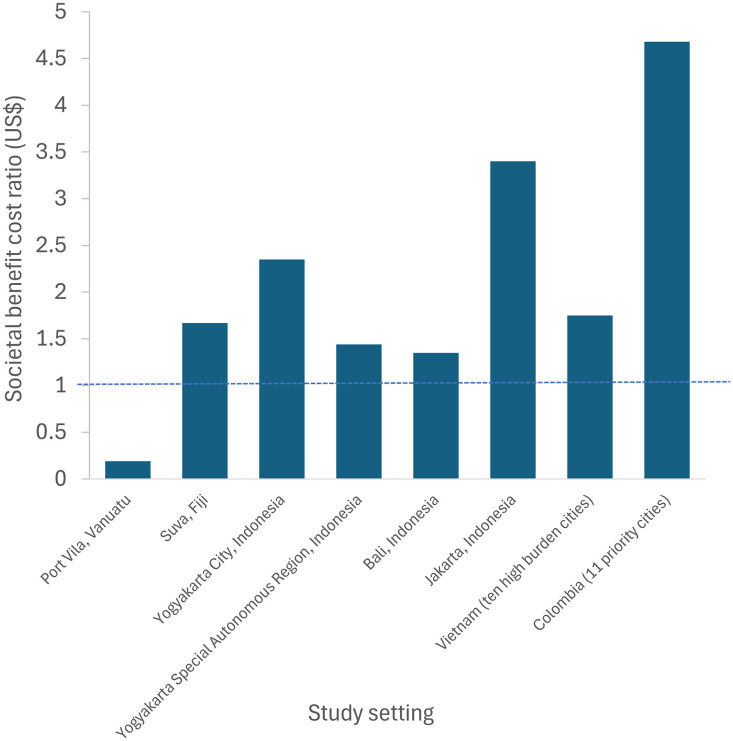
The projected societal benefit-cost ratios across different study settings. Bars show the estimated societal benefit–cost ratio for each implementation setting, reflecting the extent to which projected societal benefits outweigh programme costs. The dashed horizontal line at 1.0 indicates the break‑even point where benefits equal costs.

For the replacement strategy, the estimated societal benefit-cost ratios ranged between 0.19-4.68 (Table 3 and Fig 3). The only country setting below with a benefit-cost ratio below 1 related to Port Vila, Vanuatu, which only targeted approximately 54,000 people, and had a high corresponding cost per person covered (Fig 3). The reported economic benefits are highlighted in S1 File: S1 Table. In some cases, studies that did not disaggregate cost-effectiveness ratios by different perspectives reported disaggregated economic benefits (S1 File: S1 Table). When comparing the reported cost-effectiveness ratios to established cost-effective thresholds (such as <0.5 of the country’s per capita GDP [15,16]), it shows that overall, *Wolbachia* replacement-based programmes were found to be cost-effective or even often cost-saving when targeting high-burden cities (Table 3). The cost-effectiveness was not as promising for smaller-scale settings (releases covering approximately 50,000–200,000 people), and for such settings, the cost-effectiveness ratio is more likely to be above the economic threshold. This is consistent with the societal benefit-cost ratios results. This is likely because such settings have a notably higher cost per person covered (Table 2). How the costs for such settings could change if they were included within an expansion of a nearby larger programme requires further investigation.

When using the societal perspective, a national suppression-based strategy in Singapore was also projected to generate cost-savings, with societal benefit-cost ratios >1 [34] (Table 3 and S1 File: S1 Table). However, it had a notably higher total cost per person covered (approximately US$59 per person covered - Table 2) compared to the replacement-based interventions at a similar scale (Fig 2).

### Quality assessment

Quality assessment was conducted with the CHEQUE tool [24]. Overall, the included studies demonstrated generally sound methodological and reporting standards, with average scores reflecting acceptable adherence to CHEQUE criteria (S1 File: S2 Table). Key analytical features—such as target population, time horizon, perspective, and discount rate—were typically reported clearly across studies. However, several recurring areas requiring improvement were identified (S1 File: S1 Fig). Most notably, studies frequently lacked clear documentation of model validation procedures and often provided insufficient justification for key modelling choices and assumptions; in addition, the software used to conduct the analysis was rarely stated. Furthermore, incremental effects, incremental costs, and the corresponding ICERs were often not fully reported, limiting interpretability and hindering cross-study comparison. Additional gaps included incomplete reporting of resource use and limited summaries of the broader consequences of the interventions. Finally, equity considerations and ethical implications of the economic evaluations were generally not fully considered in the studies, highlighting a persistent gap in how dengue economic evaluations incorporate potential distributional impacts/equity considerations.

## Discussion

We identified nine studies evaluating *Wolbachia*-based interventions for dengue control. The majority (eight out of nine studies) investigated *Wolbachia* replacement-based programmes. These available economic evaluations consistently suggest that *Wolbachia*-based replacement interventions can be cost-effective for dengue control when appropriately targeted, particularly in high-density urban environments such as cities. In contrast, only one study has evaluated a suppression‑based intervention, making it difficult to draw robust conclusions about its relative cost‑effectiveness. Although the data available indicate that suppression interventions will be more costly, further research is needed to enable more informative comparisons and to examine how the cost‑effectiveness of both approaches varies across a wider range of operational and epidemiological settings.

Although the results were mostly supportive, there were important sources of variation in the projected cost-effectiveness estimates. These included:

Baseline burden: A key driver of the variation in cost-effectiveness projections was the setting’s baseline incidence of dengue infection. In settings where there was a lower baseline disease burden the *Wolbachia*-based interventions were projected to be less cost-effective. In contrast, in settings where the incidence of infection was higher, the results were more promising, and the intervention could even be cost-saving from a health sector perspective. This supports targeting the intervention to high burden areas. A caveat to this trend would be if the *Wolbachia* deployments could sustainably eliminate transmission in low transmission intensity areas. It is important to consider that, being an area-based intervention, two aspects of burden need to be considered: the dengue incidence (such as infections per 100,000 population) and the population density of that area. Shepard *et al*. [27] demonstrated that the combination of these two aspects leads to a high burden area and the most favourable benefit-cost ratios where from areas with the highest population density.

Cost and scale of the *Wolbachia* deployments: The cost per person covered was also an important driver of the projected cost-effectiveness and was heavily influenced by the scale of the deployment- generally decreasing as the targeted population increased (Fig 2). Due to the potentially high fixed costs for this intervention, if the targeted population is not large enough, the cost per person covered will be high, and the cost-effectiveness projections will be less promising. In contrast, in settings where the cost per person covered was lower, the intervention could be projected to be cost-saving. As programmes scale up and programmes implementation methods are refined, there is potential for the costs of *Wolbachia* deployments to be reduced compared to the values assumed within these studies, and the actual costs incurred for large-scale deployments across settings require further investigation [8].

Impact of *Wolbachia* deployments and duration of its effects: A key driver of the cost-effectiveness and public health impact was the assumed effectiveness of *Wolbachia* deployments in reducing the incidence of dengue infection, and for the replacement-based strategy, the duration of its impact. Regarding the latter, most studies considered 10 years of benefits in their base-case scenario. When longer impacts were considered, the estimates of the cost-effectiveness of the replacement-based strategy increased; however, there are limited data to parametrise the duration of impact at this time. It is important to acknowledge that the effectiveness of Wolbachia deployments can be heterogeneous, and outcomes in different settings could be lower than what is being assumed (further discussed in the Areas that need further research section).

Perspective and cost savings included in the cost-effectiveness ratio: One of the most significant drivers in the variation of the projected cost-effectiveness ratios was the perspective being considered. In this case, the perspective would not influence the projected cost of the intervention but could influence the cost savings included in the cost-effectiveness calculation (S1 File: S1 Box). Considering net cost-effectiveness ratios from the health sector perspective would account for savings in averted direct medical costs. In contrast, considering net cost-effectiveness ratios from the societal perspective would also include averted direct non-medical costs and the estimated monetary value of the productivity gains associated with preventing dengue cases. The cost-effectiveness ratios of the replacement strategy from a societal perspective were often negative, indicating that the economic benefits relative to the comparator outweighed the costs. Note that these “cost savings” can include non-fiscal costs. It should be noted that the projected gross cost-effectiveness ratios (which do not account for any cost savings/offsets) were less promising. Dengue is an example where the perspective can have a large influence on cost-saving estimates due to the high proportion of non-medically attended cases. This highlights the importance of using a disaggregated societal perspective [48,49], where the costs and outcomes are disaggregated, either by sector of the economy or by who incurs them, whenever possible, making it possible to interpret the results from a range of perspectives. It should be noted that the inclusion of productivity costs within cost-effectiveness ratios remains a debated area – particularly those related to averted mortality [11,50–56].

There was notable variation in how results were reported across studies. This heterogeneity limited the scope for further quantitative analysis and prevented more direct comparison of the findings. To help address these challenges, Box 1 outlines recommendations to support future cost-effectiveness analysis of *Wolbachia-*based biocontrol interventions.

**Box 1. Recommendations for future cost-effectiveness analysis of *Wolbachia-*based biocontrol interventions.**

**Setting and baseline burden of infection and disease**

- State whether the evaluation is retrospective or simulating into the future.
- Clearly report;
  - The population and area where the intervention is implemented (release area) and the population and area expected to benefit from the intervention.
  - The approach used to estimate the incidence of symptomatic infection and disease – discuss uncertainty and compare to other estimates relating to the same setting.
  - The approach to stratify the assumed incidence into the different levels of disease severity (e.g., hospitalised, outpatient, and seeking informal care).
  - The cost of illness parameters used (and their sources).

**Intervention costs and effectiveness**

- Justify the assumed effectiveness and duration of impact – exploring a wide range in the sensitivity analysis.
- Clearly report the cost per person, cost per km^2^ covered and total cost of the *Wolbachia*-based biocontrol intervention disaggregated by year of the programme. In addition, state the methodology employed to calculate these values.
- Clearly state the time horizon and the duration over which the intervention was assumed to be effective.

**Output**

- Report incremental cost-effectiveness ratios and/or net monetary benefit. Do not only report gross cost-effectiveness ratios.
- Clearly state which output relates to which perspective.
- Stratify the projected economic benefits according to the cost type/beneficiary.
- Report the breakpoint year (i.e., the year in which the projected economic benefits start to outweigh the cost of the intervention).
- Report aggregated outputs (i.e., the overall cost-effectiveness of the programme) as well as outputs stratified by the targeted areas.

### Comparison to other dengue interventions

Reviews summarising the economic evaluations of other types of dengue interventions have been conducted [57–60]. These found that other types of dengue interventions can also be cost-effective. Interestingly, an overarching review of the economic evidence of *Aedes*-borne arboviruses found that “the current economic evidence of Aedes-borne arbovirus lacks consistency on many methodological areas” [60]. This is consistent with our results.

When comparing interventions, it is important to consider not only their cost-effectiveness ratio but also their overall population-level impact on dengue. For example, it was estimated that when using the WHO’s pre-vaccination screening recommendation, the Dengvaxia vaccine would also be cost-saving when using a societal perspective [61]. However, its overall impact in terms of the reduction in hospitalisation was much more limited than that of *Wolbachia*-based interventions [61].

### Implementation and equity

This review focused on the estimated cost-effectiveness of *Wolbachia‑*based biocontrol interventions. However, real-world implementation of *Wolbachia*-based biocontrol interventions is shaped by operational feasibility, community engagement, regulatory pathways, and health‑system capacity. These factors are as critical as economic evidence when considering the adoption of this intervention. Country ownership, the availability of the technology from different sources, and financing arrangements require careful consideration to ensure programs can be scaled and maintained in an equitable manner.

The equity implications surrounding *Wolbachia‑*based biocontrol interventions are also complex. On the one hand, significantly reducing the incidence of dengue can benefit poorer populations that face high out‑of‑pocket costs associated with the disease. In addition, as an area‑based intervention that does not require any action from residents to benefit, *Wolbachia-*based interventions are expected to provide broadly equitable protection for all individuals living within the release zone, including across socio‑economic groups. However, while targeting the intervention to high‑burden urban areas can maximise health gains and economic benefits, concentrating deployment in cities risks widening geographic and socioeconomic inequities if rural and peri-urban populations are left without access to disease control. This highlights that comprehensive dengue control in endemic countries will require multiple complementary interventions and should not rely on a single solution.

### Areas that need further research

#### Projecting the burden of dengue infection and disease.

Dengue cases are notoriously underreported. However, the extent of underreporting varies considerably between and within countries. Due to this, methods are needed to account for the number of symptomatic cases occurring beyond those reported (such as those adjusting reported case numbers using expansion factors or using model-based projections). The different approaches used to estimate the actual burden of symptomatic dengue cases can yield different results [43,62,63]. If the estimated incidence of dengue is overestimated, it could subsequently overestimate the impact and cost-effectiveness of the intervention, and vice versa*.* Improvements to the methods used for reconstructing the actual disease and infection burden of dengue and better characterizing the heterogeneity in case reporting across local and global settings would be beneficial. A range of measures can be used to characterise dengue burden, and different metrics will be appropriate when assessing the suitability of areas for different types of intervention. For an area‑based intervention, it is particularly important to consider both the incidence of infection and the population density of the targeted area, as these jointly determine the actual disease burden and the potential impact of control efforts.

There is also uncertainty regarding the proportion of symptomatic dengue cases that seek formal treatment and those that require hospitalisation. Primary data related to dengue healthcare-seeking behaviour are limited, and data for other conditions/non-dengue specific data are typically used as a proxy (such as data related to the proportion of children with acute respiratory infections or fever seeking treatment at a public sector facility [64,65]). This is an area that also needs further attention, and further data from a range of settings would be beneficial. Within Turner *et al.* [29], the different scenarios related to these parameters for Vietnam had a significant impact on the results, changing the cost per DALY averted from the health sector perspective between US$420 (2020 prices) to negative (i.e., cost-saving).

Notable variation was also observed in the disability weights used to calculate the DALYs associated with dengue (values summarised in S1 File: S2 Box). The disability weights used were often based on those estimated by Zeng *et al*. [44] (Table 1). These are higher than the weights used for acute dengue illness by the GBD and were based on a systematic analysis of disability/quality of life lost from a symptomatic non-fatal dengue episode (S1 File: S2 Box). It should be noted that the inclusion of GBD assumed level of post-acute consequences (persistent symptoms) significantly increases the estimated years of healthy life lost due to dengue-related disability resulting from dengue [66,67]. Note that the DALY weights for inpatient and outpatient dengue cases estimated by Zeng *et al*. [44] were stratified, including and excluding the GBD-assumed level of post-acute consequences (S1 File: S2 Box). However, this is an area of uncertainty that requires further investigation, particularly regarding the incidence, severity, and duration of any persistent symptoms from a wider range of settings [68–70].

Further research is also needed to improve our understanding of the averted cost of illness associated with dengue cases [71].

### Capturing the effectiveness and uncertainty regarding the long-term impact

At this time, there are limited data related to the effectiveness of suppression-based strategies, with the only evaluation assuming a wide range of effectiveness between 40–80%. There is a need for further effectiveness data, particularly outside of Singapore. It is important to note that historically, Singapore has implemented one of the world’s most intensive vector control programmes, with sustained efforts that drove Aedes populations to levels [72]. While initially highly effective in suppressing transmission and dengue incidence, this also resulted in a progressively larger pool of susceptible individuals, which has been identified as a key driver of the scale of subsequent outbreaks when new serotypes or substantial viral introductions occur and requiring increasing amounts of control measures to keep transmission at the same level [72,73]. These epidemiological implications of these dynamics [73] warrant greater consideration when evaluating the projected long-term impact of *Wolbachia*‑based interventions.

Several of the evaluations of the replacement-based strategy based the assumed effectiveness of *Wolbachia* deployments on the results of a cluster randomised trial and quasi-experimental studies in Yogyakarta (with the *wMel* strain) [39,40]. There are at least three factors that can result in an underestimated efficacy from this type of trial: human movement, mosquito movement, and coupled transmission dynamics between trial arms [74]. Regarding the latter, Cavany *et al*. [74] highlighted the importance of transmission dynamic modelling in designing and interpreting future trials. A reanalysis of the Yogyakarta trial using spatiotemporally resolved data on the distribution of Wolbachia mosquitoes and the mobility of participants estimated an increased intervention efficacy to >80% [75]. In Colombia, following pilot releases in 2015–2016, staged city-wide *Wolbachia* deployments were undertaken in the cities of Bello, Medellín, and Itagüí between October 2016 and April 2022 [76,77]. A quasi-experimental study using interrupted time series analysis showed that notified dengue case incidence in the three cities declined by 95–97% compared to the prior decade [77].

That said, it is important to note that the successful dispersal of *Wolbachia*-infected mosquitoes can be heterogeneous and influenced by local environmental factors [28,78]. The effectiveness in other settings may therefore be lower than that found in the Yogyakarta study trial, and this potential variation requires further investigation. For example, an analysis of pilot releases of *wMel*-infected mosquitoes in Niterói, Brazil, estimated a 69% reduction in dengue incidence (95% confidence interval (CI): 54–79%) [79]. In contrast, a spatiotemporal modelling study of a release programme in Rio de Janeiro, Brazil, reported only a 38% (95% CI: 32–44%) reduction in dengue incidence [80]. In addition, a study utilising the *wAlbB* strain in Malaysia estimated a 40.3% (95% CI: 5.1–64.6%) reduction in dengue cases [4]. It should be noted that there is likely a gap between early measures of efficacy found within trials/pilot interventions and the real-world effectiveness of large-scale programmes. Therefore, it is important for future studies to explore a range of effectiveness values lower than the assumed baseline. It will be important that future economic evaluations in this area incorporate updated field data and become less reliant on long term projections.

A further source of uncertainty is the duration of the impact of the replacement-based strategy (which was a key driver of the projected cost-effectiveness). While field evidence from northern Australia demonstrates that *wMel*-infected mosquitoes remain effective more than a decade after their initial release, indicating long-term stability of *Wolbachia* in *Ae. aegypti* populations [81], more data are needed from a range of epidemiological settings. In addition, several factors could potentially reduce the long-term impact of *Wolbachia*-based interventions [28]. These include reinvasion by *Wolbachia* uninfected mosquitoes, evolution of viral resistance, temperature effects on viral blocking efficacy and inheritability, and selection of more virulent dengue virus strains. These aspects require further investigation.

Finally, it is important to consider that climate change is likely to expand the geographical distribution and transmission intensity of several vector-borne human infectious diseases, including dengue. This could potentially increase the baseline burden that could be averted by *Wolbachia* interventions, increasing its public health impact. On the other hand, it is also possible that climate change will have an impact on the long-term effectiveness of *Wolbachia-based* interventions [82–84]. In an analysis of this area, Vásquez *et al*. [84] concluded that this technology is generally robust to near-term (2030s) climate change. However, accelerated warming may challenge this in the 2050s and beyond.

### The cost of the intervention

Currently, there are limited published costing data related to the use of *Wolbachia* interventions. Many current primary cost estimates are based on mid-sized project sites. There is potential for the costs associated with *Wolbachia* deployments to be reduced over time through advances in mass mosquito production, economies of scale, and alternative implementation models [29]. The actual costs incurred for large-scale deployments require further investigation. An important area for further research is understanding the marginal cost of expanding existing programmes.

Brady *et al*. [28] highlighted how the costs of *Wolbachia* deployments show a relatively complex relationship with cost per person varying by population density in the release area and the scale of deployment. They found that because areas with higher human density require more mosquito release numbers per unit area (as they typically have higher natural mosquito population sizes), the cost per km^2^ covered increases as the human density increases. However, despite this, the cost per person covered will decrease because more people are covered in these high-density urban areas – which reduces the cost per person covered due to economies of scale. These relationships must be considered when projecting the cost of this intervention. There is a need to develop costing models to better account for how costs change as the intervention is scaled up within countries. These complexities in cost scaling (by both area and population covered) are a distinctive challenge associated with environmental interventions, leading to cost-effectiveness projections that are more highly context dependent.

There is also an ongoing need for the development/implementation of methods that reduce the cost of *Wolbachia*-based interventions. This is highlighted by Tiley *et al*. [8], who estimated that for a replacement-based strategy to be deployable in enough areas to make major contributions to reducing the global dengue burden by 25% (in line with 2030 WHO targets), the cost must ultimately be reduced to between US$0.24-7.63 (2020 prices) per person protected, in order to be cost-neutral from a health sector perspective. Hollingsworth *et al*. [85] recently developed a stochastic dynamic programming framework for determining optimal release schedules for *Wolbachia*-transinfected mosquitoes that balances the cost of dengue infection with the costs of rearing and releasing transinfected mosquitoes. Such an approach could help optimize programmes – reducing its cost. Novel ways to conduct the mosquito releases also need to be considered.

### Broader currently unquantified benefits

As well as the investigated averted cost of illness associated with controlling dengue, there are other potential benefits that are not being quantified. For example, dengue can lead to a loss of tourism revenue [86]. In addition, dengue outbreaks can cause congestion in intensive care wards, potentially having negative consequences on the care of patients with other conditions due to the deterioration of overall service quality. The benefits of *Wolbachia-*based interventions in reducing this have not been accounted for.

The current economic evaluations have only investigated the public health impact of *Wolbachia-*based interventions against dengue. However, *Wolbachia* infected mosquitoes are also refractory to the Zika virus, Chikungunya virus and, Yellow Fever virus [87,88]. This means that the overall public health impact of *Wolbachia-*based interventions will be larger, increasing its cost-effectiveness/value for money. The extent of this will vary across different settings and the endemicity of these diseases.

### Further evaluation of *Wolbachia-*based strategies and the combination with other interventions

We found nine studies evaluating *Wolbachia*-based interventions, eight of which related to a replacement-based strategy (mostly based on data related to the *wMel Wolbachia* strain). That said, there remains a need to evaluate *Wolbachia* replacement-based programmes in other settings as well as the use of other strains/distribution methods as they become available. There is also a need to further evaluate the cost-effectiveness of suppression-based strategies. It is also important to consider combinations of *Wolbachia* interventions. A key difference from both an operational and cost perspective is that suppression‑based *Wolbachia* strategies require ongoing releases, whereas replacement‑based strategies do not. This fundamental distinction has major implications for feasibility in LMIC settings. Implementing suppression at the same geographic scale as replacement would likely be substantially more costly. However, suppression approaches may still be suitable for targeted, localised hotspots where sustained releases can be focused on smaller areas with high transmission intensity. Further analysis is needed to understand how these methods perform operationally at different spatial scales and what cost structures emerge under real‑world implementation conditions.

Further evaluation of the use of *Wolbachia-*based interventions in combination with other preventive interventions is needed. This is particularly important as new vaccines become available [89]. In this context, it is important to consider that although *Wolbachia*-based interventions can be highly cost-effective, it does not mean that it is possible to use them everywhere across endemic areas, particularly in more rural areas. The different cost structures of *Wolbachia*-based interventions and vaccination become highly relevant here. *Wolbachia* programme costs depend primarily on the geographic area that must be covered, whereas vaccination costs scale with the number of individuals vaccinated. These structural differences have notable implications for where each intervention is likely to be most cost-effective. There is a need to investigate the combination of *Wolbachia*-based interventions and vaccination more comprehensively, considering the use of different interventions in different areas within countries (rural vs. urban areas).

### Limitations

A potential source of bias of the search strategy is that it would not capture studies published outside of the searched electronic databases (i.e., grey literature such as policy documents/reports, and non-English language publications etc.). Efforts were made to minimise this bias by searching the bibliographies of selected studies, and searching the grey literature as outlined in the S1 File. There could also be a degree of publication bias, with economic evaluations of this intervention with unfavourable results being less likely to be published

To provide overarching, high‑level conclusions, cost‑effectiveness ratios were compared against a threshold of 0.5 times a country’s per‑capita GDP. It is important to acknowledge that this type of standardised benchmark has notable limitations, particularly when applied across diverse settings. Ideally, policymakers should draw on locally appropriate thresholds that better reflect country‑specific opportunity costs, and/or budget constraints when making decisions.

The costs were adjusted for inflation using US inflation rates. It was not possible to apply a more precise inflation adjustment that accounted for the proportion of tradable versus non‑tradable (local) resources within each cost estimate. It should also be noted that the technology and approach used to implement *Wolbachia-*based interventions are advancing over time. Due to this, the older studies captured by this review are less likely to be representative of more recent/future programmes, particularly with regard to the intervention costs and the final product method used for *Wolbachia* deployment.

### Conclusion

Based on the currently available evidence, there is consensus that *Wolbachia*-based replacement interventions can be cost-effective for dengue control, when they are appropriately targeted, particularly in high-density urban environments (such as cities). For such high burden settings, the estimated net cost-effectiveness ratios in terms of the cost per DALY averted from the health sector perspective were typically below 0.5 the per capita GDP cost-effectiveness threshold. When taking the societal perspective and including the monetary value of the productivity gains associated with the averted cases, the economic benefits of a *Wolbachia*-based replacement intervention often outweigh its cost. A suppression-based strategy was also found to be cost-saving in Singapore from a societal perspective, however it had a notably higher cost per person covered compared to the replacement-based interventions.

That said, in settings where the baseline burden is not sufficiently high, this type of intervention is unlikely to be cost-effective. The exact conditions which determine which areas should be targeted will be setting specific. It is important to note that the successful dispersal of *Wolbachia*-infected mosquitoes can be heterogeneous and influenced by local environmental factors. Further studies are needed to investigate and quantify how this heterogeneity affects long-term projections of the cost-effectiveness of *Wolbachia-based* interventions across a wider range of operational and epidemiological settings.

This review focused on the cost‑effectiveness and value for money of *Wolbachia*‑based biocontrol interventions, synthesising the estimates currently available. While these economic considerations are important, they represent only one part of the decision-making landscape. Policymakers/stakeholders must also weigh a broader set of factors—including governance arrangements, operational logistics, community acceptance, and regulatory requirements—when assessing whether and how to implement these interventions. In addition, it is important to consider that there will be no single solution to controlling dengue, and it remains vital to consider/evaluate other interventions (such as new vaccines, other novel vector control methods, therapeutics, etc, as they become available).

## Supporting information

S1 File Supporting information. This file contains supporting methodical inflation as well as Supporting Tables with additional results.
(DOCX)

S2 File The PRISMA checklist. (DOCX)

S3 File Study database. A database of the extracted data, including the raw values and the values adjusted for inflation.
(XLSX)
