# Supplementary material for: The cost-effectiveness of Wolbachia-based biocontrol interventions for dengue: A scoping review of the available evidence
Source: PLoS Negl Trop Dis. 2026 Jun 1;20(6):e0014395. doi: 10.1371/journal.pntd.0014395 (PMC13245869; doi:10.1371/journal.pntd.0014395)
Supplement: S1 File — This file contains supporting methodical inflation as well as Supporting Tables with additional results. (DOCX) [file pntd.0014395.s001.docx]

**Supporting information: The cost-effectiveness of Wolbachia-based biocontrol interventions for controlling dengue: A scoping review of the available evidence**

Hugo C Turner^1^*, Trinh Manh Hung^2^, Oliver J Brady^3,4^, Raman Velayudhan^5^ Ilaria Dorigatti^1^, Hannah E Clapham^6^

^1^ MRC Centre for Global Infectious Disease Analysis, School of Public Health, Imperial College London, London, UK.

^2^ School of Public Health, Faculty of Health, Medicine and Behavioural Science, The University of Queensland, Australia

^3^ Department of Infectious Disease Epidemiology and Dynamics, Faculty of Epidemiology and Population Health, London School of Hygiene & Tropical Medicine, London, United Kingdom

^4^ Centre for Mathematical Modelling of Infectious Diseases, London School of Hygiene and Tropical Medicine, United Kingdom

^5^ Department of Control of Neglected Tropical Diseases, World Health Organization, Geneva, Switzerland.

^6^ Saw Swee Hock School of Public Health, National University of Singapore and National University Health System, Singapore, Singapore

**Search terms**

Pubmed search terms:

("dengue"[MeSH Terms] OR "dengue"[All Fields] OR "dengue s"[All Fields]) AND (“wolbachia"[MeSH Terms] OR "wolbachia"[All Fields] OR “biocontrol”[All Fields]) AND ("cost benefit analysis"[MeSH Terms] OR ("cost benefit"[All Fields] AND "analysis"[All Fields]) OR "cost benefit analysis"[All Fields] OR ("economic"[All Fields] AND "evaluation"[All Fields]) OR "economic evaluation"[All Fields] OR "cost effectiveness analysis"[MeSH Terms] OR ("cost effectiveness"[All Fields] AND "analysis"[All Fields]) OR "cost effectiveness analysis"[All Fields] OR ("cost"[All Fields] AND "effectiveness"[All Fields]) OR "cost effectiveness"[All Fields] ("cost"[All Fields] AND "utility"[All Fields] AND "analysis"[All Fields]) OR "cost utility analysis"[All Fields] OR "economics"[MeSH Subheading] OR "economics"[All Fields] OR “cost consequence analysis”[All Fields] OR “cost minimisation analysis”[All Fields])

OVID search terms:

(dengue*) AND (Wolbachia* OR "biocontrol" ) AND (("cost benefit" AND "analysis") OR "cost benefit analysis*” OR ("economic" AND "evaluation") OR "economic evaluation” OR ("cost effectiveness” AND "analysis") OR "cost effectiveness analysis" OR ("cost" AND "effectiveness") OR "cost-effectiveness” OR ("cost" AND "utility" AND "analysis") OR "cost utility analysis" OR "economic*" OR "economics” OR "cost consequence analysis" OR "cost minimisation analysis")

Gray literature search:

We searched a set of grey‑literature platforms and institutional repositories using simplified versions of our core search terms, depending on the source (“Wolbachia”, “dengue”, “economic evaluation”, “cost‑effectiveness”). The following sources were screened:

- World Mosquito Program (WMP) website and contract with their team
- WHO Institutional Repository for Information Sharing (IRIS)
- Google Scholar (first 200 results, sorted by relevance)
- MedRxiv and BioRxiv preprint servers. Note a pre-print would only be included if the corresponding peer-reviewed full text was not available.
- We searched for theses and dissertations via ProQuest
- Selected international HTA repositories, including:
  - INAHTA database
  - HITAP (Thailand)

Note that these searches were updated during the peer review process, but the same timeframe was considered for the identification of studies to include. Based on these grey literature searches, an additional four article where included for screening – three of which were included in the final review.

| Database search | Search terms | Results found | Additional studies included for review |
| --- | --- | --- | --- |
| Google | Dengue AND (Wolbachia) AND (cost-effectiveness OR economic evaluation) | 117 | 2 |
| International HTA database (INAHTA)  <https://database.inahta.org/> | (dengue) AND ((wolbachia) OR (biocontrol)) AND ((cost-effectiveness) OR (cost-utility) OR (cost-benefit) OR (economic evaluation) OR (cost-minimization) OR (cost consequences))) | 0 | 0 |
| World Mosquito Program website  <https://www.worldmosquitoprogram.org/> | cost-effectiveness OR economic evaluation | 2 | 0 from the website  1 shared via contact with their team |
| Health Intervention and Technology Assessment Program, Thailand  <https://www.hitap.net/> | Dengue  Wolbachia | 0 | 0 |
| Medrxiv and Biorxiv | full text or abstract or title "dengue AND wolbachia AND (cost-effectiveness OR Cost Benefit OR economic evaluation)" | 22 | 1 |
| WHO Institutional Repository for Information Sharing | dengue AND (wolbachia OR biocontrol) AND (cost-utility OR cost-effectiveness OR cost-benefit OR economic evaluation OR cost minimization OR cost consequence) | 8 (Not related to Wolbachia) | 0 |
| [ProQuest dissertations and theses global](https://search.library.uq.edu.au/discovery/dbfulldisplay?docid=alma991010830599703131&context=L&vid=61UQ_INST:61UQ&lang=en&adaptor=Local%20Search%20Engine&tab=jsearch_slot&query=any%2Ccontains%2CProQuest%20dissertations%20and%20theses%20global.&offset=0&databases=any,ProQuest%20dissertations%20and%20theses%20global.) | *dengue AND (wolbachia OR biocontrol) AND (cost-effectiveness OR (benefit-cost analysis) OR (cost-utility analysis) OR (economic evaluation) OR (cost and consequences) OR cost-minimization) AND (health economics)* | 164 | 0 |

| **S1 Box: The influence of the chosen perspective**  Net/incremental cost-effectiveness ratios evaluate the additional cost and additional outcomes of a strategy compared to the next best comparable option (in most cases for these studies the status quo comparator). Within these, the relevant cost savings relative to the comparator are deducted from the cost of the intervention (such as averted costs associated with prevented hospitalised cases). Which cost offsets/savings are included depends on the perspective of the analysis:   - Under the healthcare provider perspective only savings related to averted direct medical costs that are incurred by the healthcare providers and averted costs related to the government’s current dengue prevention and control activities costs are considered. - Under the health sector perspective: only savings related to averted direct medical costs (from both the patients and the healthcare providers) and averted costs related to the government’s current dengue prevention and control activities costs are considered. - Under the societal perspective: in addition to the savings in averted direct medical costs, the savings related to the patients’ prevented direct non-medical costs (such as transport to the hospital/clinic) and the estimated monetary value of the prevented productivity losses that would have been associated with a dengue case. Due to ongoing debates in this area, the results from the societal perspective can be shown both including and excluding the productivity gains related to prevented premature mortality. |
| --- |

| **S2 Box: Summary of the disability weights used for dengue – adapted from [1]**  GBD 1990 (1996 revision)  The first disability weight for dengue was outlined within the 1996 revision of GBD 1990. This only captured dengue hemorrhagic fever with an assumed average duration of 30 days and a corresponding disability weight of 0.2 [2, 3].  Meltzer *et al.* [4]  In a study in 1998 (before the subsequent GBD update), Meltzer *et al.,* [4] used a higher disability weight of 0.81 for all symptomatic dengue cases but assumed shorter durations of illness (based on clinical data): Dengue fever, dengue with severe manifestations (but not requiring hospitalization) and hospitalized cases were assumed to have an average duration of 4 days, 10 days and 14 days, respectively [4]. The 0.81 disability weight was based on the disability scores within the class five severity level defined within the original GBD study commissioned by the World Bank (“*needs assistance with instrumental activities of daily living such as meal presentation, shopping or housework*”) [4, 5]. Subsequent studies have also used this higher disability weight, though the assumed duration of illness varied [1].  GBD 2004 update  Within the GBD 2004 update, both dengue fever and dengue hemorrhagic fever/dengue shock syndrome were included:   - 94% of the symptomatic cases were assumed to have dengue fever with a mean duration of 5.5 days and were assigned a disability weight of 0.21 [2, 6]. - 6% of the symptomatic cases were assumed to have dengue hemorrhagic fever/dengue shock syndrome with an average duration of 11 days and were assigned a disability weight of 0.5 [2, 6].   Post GBD 2013  Within the GBD 2013 symptomatic dengue cases are assigned into two acute health states:   - 94.5% were assigned the disability weight for a moderate acute infectious disease episode (0.051) with a mean duration of six days [7, 8]. - 5.5% were assigned the disability weight for a severe acute infectious disease episode (0.133) with a mean duration of 14 days [7, 8].   In addition, 8.4% of the dengue symptomatic cases were assumed to have post-dengue chronic fatigue and assigned the disability weight for “infectious disease-post-acute consequences” (of 0.219), with a mean duration of six months.  Zeng *et al*. [9]  Zeng *et al*. [9] performed a systematic analysis of disability/quality of life lost from a symptomatic non-fatal dengue episode (based on a combination of a systematic literature review, statistical modelling, and probabilistic sensitivity analyses). These accounted for the disability occurring during the onset, recovery, and persistent-symptom phases of a non-fatal dengue episode.  - YLD per ambulatory episode: 0.0307 (0.0107 not including persistent symptoms i.e. acute only)  - YLD per hospitalized episode: 0.0351 (0.0152 not including persistent symptoms i.e. acute only) |
| --- |
| *GBD: Global burden of disease. YLD: Years lived with a disability*  *Details for* *GBD 2010 are not available.* |

| **S1 Table: Estimated economic benefit of the intervention stratified by the perspective** | | | | |
| --- | --- | --- | --- | --- |
| **Study** | **Setting** | **Economic benefit (US$ millions) - health care provider or payer perspective (adjusted to 2024 prices))** | **Economic benefit (US$ millions) - health sector perspective (adjusted to 2024 prices))** | **Economic benefit (US$ millions) – societal perspective (adjusted to 2024 prices)** |
| Soh *et al.* 2021 | Singapore | - | - | 40% effectiveness: 274.94 (absolute value) |
|  |  |  |  | 80% effectiveness: 787.24 (absolute value) |
| Brady *et al.* 2020 | Yogyakarta City, Indonesia | - | 0.56 (95% CI: 0.17–1.45) per year (absolute value) | 1.19 (95% CI: 0.43-2.63) per year (absolute value) |
|  | Yogyakarta Special Autonomous Region, Indonesia | - | 2.62 (95% CI: 0.81–5.70) per year (absolute value) | 5.49 (95% CI: 2.09 -10.90) per year (absolute value) |
|  | Jakarta, Indonesia | - | 15.67 (95% CI: 5.45–30.43) per year (absolute value) | 32.86 (95% CI: 12.84-59.88) per year (absolute value) |
|  | Bali, Indonesia | - | 3.13 (95% CI: 1.00–6.20) per year (absolute value) | 6.49 (95% CI: 2.49-11.95) per year (absolute value) |
| Turner *et al*. 2023 | Vietnam (ten high burden cities) | 74.50 (absolute value) | 150.82 (absolute value) | 421.49 (absolute value) |
| Shepard *et al*. 2020 | Suva, Fiji | - | 1.33 (absolute value) | 5.46 (absolute value) |
|  | Port Vila, Vanuatu | - | 0.06 (absolute value) | 0.55 (absolute value) |
| Barbosa *et al.* 2023 | State of Goiás, Brazil | Not reported | - | 21.72 (net relative to the comparator) |
| Zimmermann *et al*. 2024 | Brazil (Seven priority cities) | Overall value not reported – results for individual cites shown in the studies' appendix | Overall value not reported – results for individual cites shown in the studies appendix | 5 years effect: 0.13 per 1000 (net relative to the comparator) |
|  |  |  |  | 10 years effect: 0.29 per 1000 (net relative to the comparator) |
|  |  |  |  | 20 years effect: 0.55 per 1000 (net relative to the comparator) |
| Shepard *et al*. 2024 (and Shepard *et al*. 2025) | Colombia (11 priority cities) | - | 96.03 (absolute value) | 241.00 (absolute value) |
| Knerer *et al*. 2020 | Thailand | Not explicitly reported | - | Not explicitly reported |
| Suwantika *et al.* 2020 | Indonesia | Wol+Vaccination: 17.71 (absolute value) | Wol+Vaccination: 29.48 (absolute value) | - |
| *Wol: Wolbachia*  *Absolute values do not account for the cost of the intervention*  *^1^Based on the summed reported costs averted by year. A discount rate of 3% was applied to the values.* | | | | |

| **S2 Table: Results of the quality assessment.** | | | | | | | | | | | | | |
| --- | --- | --- | --- | --- | --- | --- | --- | --- | --- | --- | --- | --- | --- |
|  | **Methods** | | | | | |  | **Reporting** | | | | | |
| **Study** | **NA = 1** | **Max Score** | **% of Max Score** | **NA = NA** | **Max Score NA = NA** | **% of Max Score NA** |  | **NA = 1** | **Max Score** | **% of Max Score** | **NA = NA** | **Max Score NA = NA** | **% of Max Score NA** |
| Knerer *et al.* (2020) | 76.00 | 100.00 | 76.00 | 74.00 | 98.00 | 75.51 |  | 69.50 | 100.00 | 69.50 | 65.50 | 96.00 | 68.23 |
| Zimmerman *et al.* (2024) | 92.00 | 100.00 | 92.00 | 90.00 | 98.00 | 91.84 |  | 94.00 | 100.00 | 94.00 | 90.00 | 96.00 | 93.75 |
| Soh *et al.* (2021) | 80.50 | 100.00 | 80.50 | 78.50 | 98.00 | 80.10 |  | 80.50 | 100.00 | 80.50 | 76.50 | 96.00 | 79.69 |
| Turner *et al.* (2023) | 86.50 | 100.00 | 86.50 | 84.50 | 98.00 | 86.22 |  | 81.50 | 100.00 | 81.50 | 77.50 | 96.00 | 80.73 |
| Barbosa *et al.* (2023) | 69.00 | 100.00 | 69.00 | 69.00 | 100.00 | 69.00 |  | 75.50 | 100.00 | 75.50 | 71.50 | 96.00 | 74.48 |
| Suwantika *et al.* (2020) | 67.50 | 100.00 | 67.50 | 67.50 | 100.00 | 67.50 |  | 75.00 | 100.00 | 75.00 | 71.00 | 96.00 | 73.96 |
| Shepard *et al.* (2020) | 69.00 | 100.00 | 69.00 | 67.00 | 98.00 | 68.37 |  | 70.50 | 100.00 | 70.50 | 66.50 | 96.00 | 69.27 |
| Shepard *et al.* (2025) | 86.50 | 100.00 | 86.50 | 84.50 | 98.00 | 86.22 |  | 87.00 | 100.00 | 87.00 | 83.00 | 96.00 | 86.46 |
| Brady *et al.* (2020) | 88.00 | 100.00 | 88.00 | 86.00 | 98.00 | 87.76 |  | 96.00 | 100.00 | 96.00 | 92.00 | 96.00 | 95.83 |
| *Generated using the R code generated by Dijk et al. [10].* | | | | | | | | | | | | | |


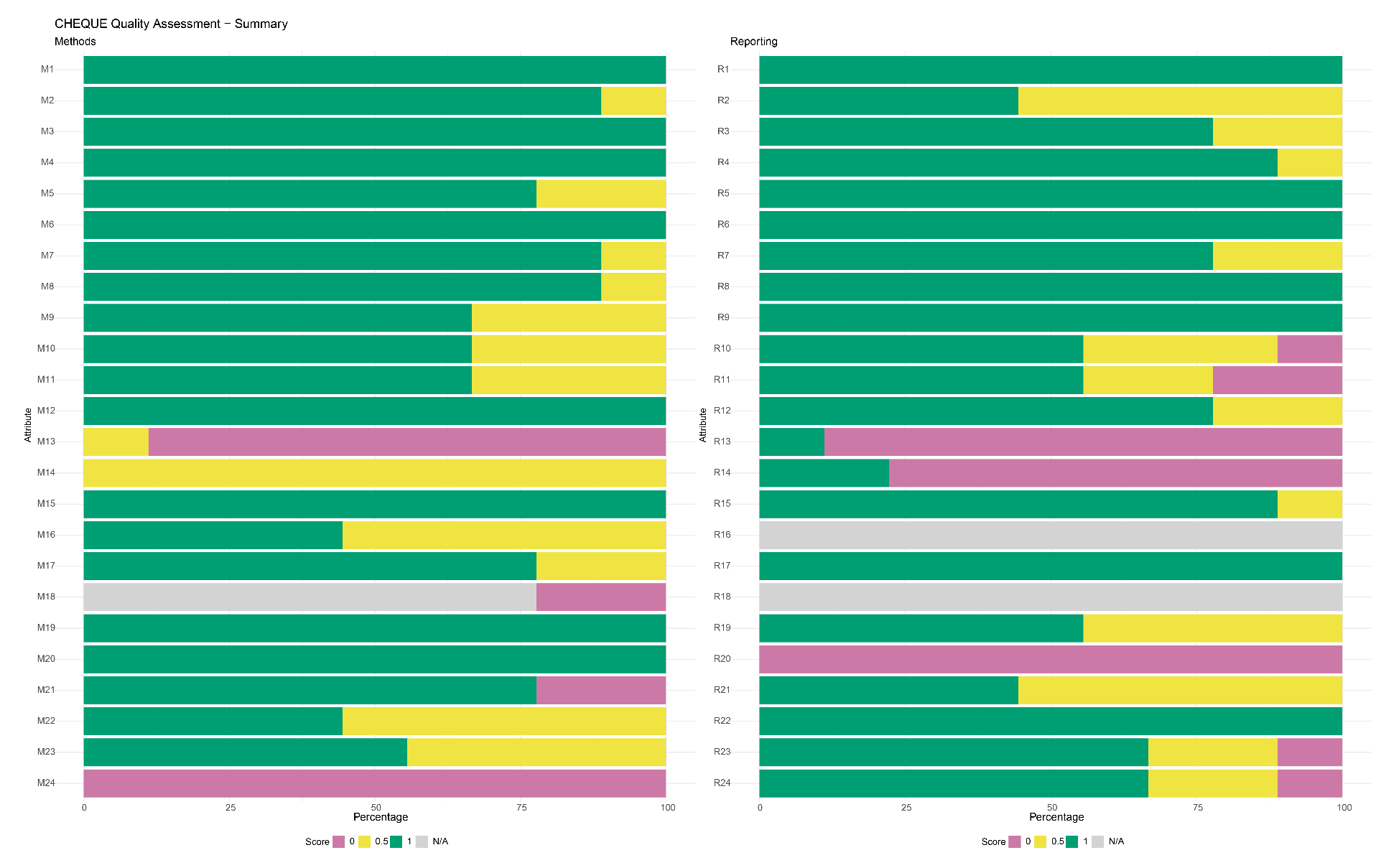


**S1 Fig: Summary of the quality assessment.** *Figure was generated using the R code generated by Dijk et al. [10].*

**Reference**

1. Hung TM, Clapham HE, Bettis AA, Cuong HQ, Thwaites GE, Wills BA, et al. The Estimates of the Health and Economic Burden of Dengue in Vietnam. Trends Parasitol. 2018;34(10):904–18. Epub 2018/08/14. doi: 10.1016/j.pt.2018.07.007. PubMed PMID: 30100203; PubMed Central PMCID: PMCPMC6192036.

2. Beatty ME, Beutels P, Meltzer MI, Shepard DS, Hombach J, Hutubessy R, et al. Health economics of dengue: a systematic literature review and expert panel's assessment. Am J Trop Med Hyg. 2011;84:473–88. doi: 10.4269/ajtmh.2011.10-0521. PubMed PMID: 21363989.

3. Mathers CD, Ezzati M, Lopez AD. Measuring the burden of neglected tropical diseases: the global burden of disease framework. PLoS Negl Trop Dis. 2007;1(2):e114. doi: 10.1371/journal.pntd.0000114.

4. Meltzer MI, Rigau-Perez JG, Clark GG, Reiter P, Gubler DJ. Using disability-adjusted life years to assess the economic impact of dengue in Puerto Rico: 1984-1994. Am J Trop Med Hyg. 1998;59(2):265–71. doi: 10.4269/ajtmh.1998.59.265.

5. Murray CJ. Quantifying the burden of disease: the technical basis for disability-adjusted life years. Bull World Health Organ. 1994;72(3):429–45.

6. Mathers C, Fat DM, Boerma JT. The global burden of disease: 2004 update: World Health Organization; 2008.

7. Salomon JA, Haagsma JA, Davis A, de Noordhout CM, Polinder S, Havelaar AH, et al. Disability weights for the Global Burden of Disease 2013 study. Lancet Global Health. 2015;3:e712–e23. doi: 10.1016/S2214-109X(15)00069-8.

8. Stanaway JD, Shepard DS, Undurraga EA, Halasa YA, Coffeng LE, Brady OJ, et al. The global burden of dengue: an analysis from the Global Burden of Disease Study 2013. Lancet Infect Dis. 2016;16:712–23. doi: 10.1016/S1473-3099(16)00026-8.

9. Zeng W, Halasa-Rappel YA, Durand L, Coudeville L, Shepard DS. Impact of a Nonfatal Dengue Episode on Disability-Adjusted Life Years: A Systematic Analysis. The American journal of tropical medicine and hygiene. 2018;99(6):1458–65. doi: 10.4269/ajtmh.18-0309. PubMed PMID: 30277202.

10. Dijk SW, Essafi S, Hunink MGM. An Application of the Checklist for Health Economic Quality Evaluations in a Systematic Review Setting. Value Health. 2025;28(2):250–9. doi: <https://doi.org/10.1016/j.jval.2024.10.3853>.
