# Supplementary material for: The cost-effectiveness of Wolbachia-based biocontrol interventions for dengue: A scoping review of the available evidence
Source: PLoS Negl Trop Dis. 2026 Jun 1;20(6):e0014395. doi: 10.1371/journal.pntd.0014395 (PMC13245869; doi:10.1371/journal.pntd.0014395)
Supplement: Attachment — Submitted filename: Wol health econ Comments.docx [file pntd.0014395.s004.docx]

We would like to thank the reviewers and editors for their thoughtful comments and the time they have dedicated to considering our article. Their feedback has significantly improved the clarity and quality of the manuscript. Please find our detailed responses below:

**Reviewers' Comments:**


**Methods**


 Reviewer #1: See comments

Reviewer #2: (No Response)

Reviewer #3: The manuscript acknowledges that as a scoping review, it did not assess the risk of bias or methodological quality of the included studies. This is a major limitation for a review aiming to summarize and compare heterogeneous economic outcomes: without quality assessment, it is impossible to weigh how much to rely on each estimate. An assessment should be added and used to stratify or nuance the conclusions.

Response: In line with established methodological guidance scoping reviews are not required to undertake a formal risk‑of‑bias or methodological quality assessment, as their primary purpose is to map the breadth and nature of the evidence rather than to evaluate the certainty or comparative reliability of individual estimates. Nonetheless, we agree that providing an indication of study quality in this context will add interpretive value, particularly when summarising heterogeneous economic outcomes. We have therefore added quality assessment using the CHEQUE tool.

**Methods - Quality assessment**

*Quality assessment of the identified relevant studies was undertaken using the CHEQUE tool {Kim, 2023 #355}. This was conducted by two independent reviewers (TMH and HCT) following the procedures outlined for screening and data extraction.*

**Results - Quality assessment**

*Quality assessment was conducted with the CHEQUE tool [24]. Overall, the included studies demonstrated generally sound methodological and reporting standards, with average scores reflecting acceptable adherence to CHEQUE criteria (Supporting Table S2). Key analytical features—such as target population, time horizon, perspective, and discount rate—were typically reported clearly across studies. However, several recurring areas requiring improvement were identified (Supporting Figure S2). Most notably, studies frequently lacked clear documentation of model validation procedures and often provided insufficient justification for key modelling choices and assumptions; in addition, the software used to conduct the analysis was rarely stated. Furthermore, incremental effects, incremental costs, and the corresponding ICERs were often not fully reported separately, limiting interpretability and hindering cross-study comparison. Additional gaps included incomplete reporting of resource use and limited summaries of the broader consequences of the interventions. Finally, equity considerations and ethical implications of the economic evaluations were generally not addressed, highlighting a persistent gap in how dengue economic evaluations incorporate potential distributional impacts/equity considerations.*

The lack of a registered protocol (e.g., in PROSPERO) reduces transparency and opens the door to selection and reporting bias. I recommend registering the protocol or justify the decision to the editor.

Regarding protocol registration, we appreciate the reviewer’s concern about transparency. This review was designed as a relatively simple methodological scoping exercise, and at the time of initiation, we did not judge formal protocol registration to be necessary compared to something more complex like a systematic review of the effectiveness of an intervention, where it would be highly important. Also to our knowledge PROSPERO does not accept registration of scoping reviews. We have added the following to the methods and will discuss further with the editor:

*Because this scoping review was undertaken as an iterative exploratory exercise intended to map the emerging evidence, no protocol was registered at the time the review was initiated.*

Limits of the bibliographic review. Why do you not have search in Web of science?

Our initial search strategy focused on databases most commonly used for economic and public health evidence (MEDLINE (via OVID), Embase Classic+Embase (via OVID), Global Health - OVID, PubMed, and Econ Lit), which we judged to provide comprehensive coverage for the scope of this review.

Why not including non-english articles?

We agree that this is a limitation (and have that listed in our limitations). Resource constraints meant that it was not possible to directly include non-English articles. This is a common limitation of such reviews. That said, none of the studies assessed for eligibility were excluded for this reason.

The manuscript acknowledges incomplete capture of grey literature and reports contact with the World Mosquito Program and the use of Google Scholar. However, details are lacking about this opportunistic research. Please complete. Were institutional repositories, NGO reports, theses, ministry reports, or preprints searched? I suggest expanding the grey literature search and documenting the results.

Response: We agree that it would be useful to strengthen the grey literature search. We have extended this and added the following to the appendix

We have added the following to the appendix.

*We searched a set of grey‑literature platforms and institutional repositories using simplified versions of our core search terms, depending on the source (“Wolbachia”, “dengue”, “economic evaluation”, “cost‑effectiveness”). The following sources were screened:*

- *World Mosquito Program (WMP) website and contract with their team*
- *WHO IRIS (Institutional Repository for Information Sharing)*
- *Google Scholar (first 200 results, sorted by relevance)*
- *MedRxiv and BioRxiv preprint servers. Note a pre-print would only be included if the corresponding peer-reviewed full text was not available.*
- *We searched for theses and dissertations via ProQuest*
- *Selected international HTA repositories, including:*
  - *INAHTA database*
  - *HITAP (Thailand)*

*Note that these searches were updated during the peer review process but the same timeframe was considered for the identification of studies to include.*

Based on these grey literature searches, an additional four article where included for screening – three of which were included in the final review

| **Database search** | **Search terms** | **Results found** | **Additional studies included for review** |
| --- | --- | --- | --- |
| Google | Dengue AND (wolbachia) AND (cost-effectiveness OR economic evaluation) | 117 | 2 |
| International HTA database (INAHTA)  <https://database.inahta.org/> | (dengue) AND ((wolbachia) OR (biocontrol)) AND ((cost-effectiveness) OR (cost-utility) OR (cost-benefit) OR (economic evaluation) OR (cost-minimization) OR (cost consequences))) | 0 | 0 |
| World Mosquito Program website  <https://www.worldmosquitoprogram.org/> | cost-effectiveness OR economic evaluation | 2 | 0 from the website  1 shared via contact with their team |
| Health Intervention and Technology Assessment Program, Thailand  <https://www.hitap.net/> | Dengue  Wolbachia | 0 | 0 |
| Medrxiv and Biorxiv | full text or abstract or title "dengue AND wolbachia AND (cost-effectiveness OR Cost Benefit OR economic evaluation)" | 22 | 1 |
| WHO Institutional Repository for Information Sharing | dengue AND (wolbachia OR biocontrol) AND (cost-utility OR cost-effectiveness OR cost-benefit OR economic evaluation OR cost minimization OR cost consequence) | 8 (Not related to Wolbachia) | 0 |
| [ProQuest dissertations and theses global](https://search.library.uq.edu.au/discovery/dbfulldisplay?docid=alma991010830599703131&context=L&vid=61UQ_INST:61UQ&lang=en&adaptor=Local%20Search%20Engine&tab=jsearch_slot&query=any%2Ccontains%2CProQuest%20dissertations%20and%20theses%20global.&offset=0&databases=any,ProQuest%20dissertations%20and%20theses%20global.) | *dengue AND (wolbachia OR biocontrol) AND (cost-effectiveness OR (benefit-cost analysis) OR (cost-utility analysis) OR (economic evaluation) OR (cost and consequences) OR cost-minimization) AND (health economics)* | 164 | 0 |

**Results**

Reviewer #1: See comments

Reviewer #2: (No Response)

Reviewer #3: The current review lists the results but does not provide a structured critical analysis explaining how differences in: how diseases were measured, disability weights used, whether vector control costs were included, the timeframes studied, and the assumptions about the intervention's effectiveness and duration affect the direction and size of the cost-effectiveness ratios.
In addition to descriptive tables, numerical analysis to answer relevant question. It would add depth and robustness to perform more complete analyses and figures that could be derived from the data extracted from the papers.

Response: We have also added new figures to summarize the key trends more clearly.

| Populuation covered - millions | Cost per person covered |
| --- | --- |
| 0.46 | 15.4094 |
| 2.14 | 17.4236 |
| 11.19 | 14.463 |
| 2.44 | 25.735 |
| 20.02 | 10.163 |
| 0.225 | 14.6814 |
| 0.054 | 53.9937 |
| 22.12 | 2.62241 |
| 8.34 | 5.61579 |

**Figure 2: The reported cost per person covered compared to the population covered.** Labels next to each point indicate the population size for that setting. Only costs related to primary data were considered (hypothetical cost benchmarks/targets were not plotted). Uses the data related to the accelerated scenario for Brady *et al.* [25].

| Study setting | Societal benefit cost ratio (US$) |
| --- | --- |
| Port Vila, Vanuatu | 0.19 |
| Suva, Fiji | 1.67 |
| Yogyakarta City, Indonesia | 2.35 |
| Yogyakarta Special Autonomous Region, Indonesia | 1.44 |
| Bali, Indonesia | 1.35 |
| Jakarta, Indonesia | 3.4 |
| Vietnam (ten high burden cities) | 1.75 |
| Colombia (11 priority cities) | 4.68 |

**Figure 3: The projected societal benefit cost ratios across different study settings**. Bars show the estimated societal benefit–cost ratio for each implementation setting, reflecting the extent to which projected societal benefits outweigh programme costs. The dashed horizontal line at 1.0 indicates the break‑even point where benefits equal cost.

We fully agree that additional quantitative analyses would, in principle, add depth and be highly valuable. However, the reporting and methodological approaches across the included studies were highly heterogeneous (including the type of cost-effectiveness ratio calculated This level of heterogeneity limits the feasibility and validity of conducting structured quantitative analyses to isolate the impact of individual assumptions. For the same reason, a formal meta‑analysis was not appropriate. Due to this, we have a box explicitly highlighting recommendations for figure studies to improve consistency that would allow such analysis in the future. We have added the following into the highlight this point

*Results:*

*The cost-effectiveness ratios stratified by the different perspectives employed are summarised in Table 3 (with the corresponding economic benefits reported in Supporting Information file 1: Supporting Table S1). For this intervention, the broader the perspective, the lower the cost-effectiveness ratio. This is because the broader the perspective the higher the averted cost of illness associated with dengue cases; with the societal perspective including the estimated monetary value of the prevented productivity losses that would have been associated with a dengue case. For the same reason, the projections based on gross cost‑effectiveness ratios (ignoring all cost offsets) appeared less favourable then ICER based output. Differences in the chosen analytic perspective as well as the types of ratios reported, made further direct comparison across studies challenging. As a result, further quantitative synthesis of the cost‑effectiveness*

*Discussion:*

*There was notable variation in how results were reported across studies. This heterogeneity limited the scope for further quantitative analysis and prevented more direct comparison of the findings. To help address these challenges, Box 1 outlines recommendations to support future cost-effectiveness analysis of Wolbachia-based biocontrol interventions.*

**Conclusions**

Reviewer #1: See comments

Reviewer #2: (No Response)

Reviewer #3: Lack of discussion on equity and operational feasibility: Cost-effectiveness alone is insufficient — governance, logistics, quality of the implementation, durability, program sustainability, community acceptance, regulatory issues, and equity considerations (e.g., urban vs. rural disparities or low vs high income areas) are not examined in depth.
I would also encourage the authors to address the broader issue of accessibility and democratization of the Wolbachia wMel technique. At present, implementation and large-scale deployment are primarily coordinated by a single established private organization, being accesible only for the governments or communities that can be pay this program. Therefore, it may limit transparency, competition, and the ability of countries to independently adopt or adapt the technology. For long-term sustainability and equitable global access, discussing this dimension is relevant. The potential conflicts of interest of reviewers might be more clearly stated, as in this meta-analysis, at least on of the authors receive funding for WMP, please be as transparente as possible.

Response: We fully agree that cost-effectiveness alone is not sufficient when making policy decisions and other factors have to be considered (such as within an HTA process). We have added a dedicated section in the discussions on the equity and operational aspects.

Implementation and equity

*This review focused on the estimated cost-effectiveness of Wolbachia‑based biocontrol interventions. However, real-world implementation of Wolbachia-based biocontrol interventions is shaped by operational feasibility, community engagement, regulatory pathways, and health‑system capacity. These factors are as critical as economic evidence when considering the adoption of this intervention. Country ownership, the availability of the technology from different sources, and financing arrangements require careful consideration to ensure programs can be scaled and maintained in an equitable manner.*

*The equity implications surrounding Wolbachia‑based biocontrol interventions are also complex. On the one hand, significantly reducing the incidence of dengue can benefit poorer populations that face high out‑of‑pocket costs associated with the disease. However, while targeting the intervention to high‑burden urban areas can maximise health gains and economic benefits, concentrating deployment in cities risks widening geographic and socioeconomic inequities if rural and peri-urban populations are left without access to disease control. This highlights that comprehensive dengue control in endemic countries will require multiple complementary interventions and should not rely on a single solution.*

Please note that while we fully agree regarding the importance of these broader factors, it is not possible to fully account for all of the aspects you mentioned in one review paper. We have also added the following to the conclusion to highlight these factors prominently.

*This review focused on the cost‑effectiveness and value for money of Wolbachia‑based biocontrol interventions, synthesising the estimates currently available. While these economic considerations are important, they represent only one part of the decision-making landscape. Policymakers/stakeholders must also weigh a broader set of factors—including governance arrangements, operational logistics, community acceptance, and regulatory requirements—when assessing whether and how to implement these interventions.*

To make the COI as transparent as possible, we have added the following to the methods.

*“As stated in the relevant conflict of interest section, it is important to note that HCT has received research funding from The World Mosquito Program in the past. The World Mosquito Program had no involvement in this paper (other than being a potential source of studies within the search of the grey literature).”*

**Editorial and Data Presentation Modifications?**


Reviewer #1: Specific comments
P6, para 1 Suggest changing “These rely primarily on vector control” to “These rely primarily on vector control and vaccination.” Note: this revision promotes consistency with the finding in results (paragraph 2) that examines the combination of Wolbachia and vector control.

Response: We have edited accordingly

Currently, these strategies are predominantly reliant on vector control and increasingly vaccination.

P6, para 1 After “less likely to transmit infection…” add comma there and after closing parenthesis

Response: Had added as suggested.

P10, Results, last paragraph. The authors may wish to change the phrase “above the effective threshold” to “above the economic threshold.”

Response: Have edited accordingly.

P20 ref 13. For this and other references not in journals, provide further details (e.g. specifics and links of conference presentation and/or the abstract)

Response: We have added further details where possible. Note that ref 13 is an unpublished report and therefore it is not possible to add much further information. We have made this clearer

Supplementary materials, Table 1, see general comments.

Reviewer #2: Minor comments:

In the introduction – the last sentence in the second paragraph ‘A key difference from an operational and cost perspective is that the suppression-based strategy requires ongoing releases, whereas the replacement-based strategy does not’ should go in the methods, where the stratification of the different perspectives employed is described.

Response: We thank the reviewer for this suggestion. We have removed the sentence from the introduction. We did not relocate it to the Methods section because the term “perspective” in that sentence was being used in a general descriptive sense to contrast operational features of suppression versus replacement strategies, rather than the analytic perspectives applied in the economic evaluation (in the methods).

In the methods- I was a little bit confused as to why authors state that ‘No date or language restrictions were applied to the searches’ but then non-english publications were excluded at the extraction phase, particularly considering that trials have been implemented in Latin American countries.

Response: We did not apply language limits at the search stage because we wanted a comprehensive view of the available literature and to assess whether a substantial body of relevant non‑English evidence existed. This approach ensured that potentially important studies—particularly from regions such as Latin America—were not inadvertently missed.

Non‑English publications would have been excluded at the data‑extraction stage solely because the team did not have the capacity to translate full texts. That said, no studies were ultimately excluded for this reason, as all potentially eligible full‑text articles identified through the searches were available in English. We have modified the text:

*Although no language restrictions were applied at the search stage, non‑English full texts were excluded during screening because translation resources were not available. Reviews/systematic reviews, intervention costing studies, and conference abstracts were also excluded.*

Clarify in the inclusion/exclusion criteria that pre-prints are being included in the analysis.

Response: We have added the following:

*Based on the database and grey literature searches (the latter including pre-prints), we included studies that conducted an economic evaluation (i.e. cost-utility analysis, cost-effectiveness analysis, or cost-benefit analysis [10]) related to the use of the Wolbachia-based intervention to control dengue (both suppression and replacement-based strategies). Non-English publications, reviews/systematic reviews, and conference abstracts were excluded. Intervention costing studies and economic evaluations investigating only other preventive dengue interventions were considered outside the scope of this study and were excluded.*

Define early on what is meant by smaller scale and make this categorization explicit in Table 2. Currently it’s not easy to determine how many studies were based on small areas, and how many on larger areas, which is critical to put the author’s conclusion (regarding cost-effectiveness in large urban settings) in context for the readers.

Response: We have added the following to the methods and a new column into Table 2.

*We grouped the scale of the intervention into three categories: National, large scale (targeting more than 200,000 people), small scale (targeting under 200,000 people).*

Check the typos: ‘high burden cites’ instead of cities throughout Table 1-3.

Response: Thank you for spotting this. We have corrected these and carefully checked for other typos.

In first line of conclusion, sentence missing a verb: ‘when they appropriately targeted’.

Response: We have corrected this to *“when they are appropriately targeted”*

For a better alignment with the journal scope: In the Discussion section, add a short subsection explicitly on equity and implementation; according to manuscript findings on cost-effectiveness, who would stand to be left out from cost-effectiveness-based decisions (e.g. rural poor), and what that implies for comprehensive dengue control strategies in endemic countries.

Response: We have added the following section:

Implementation and equity

*This review focused on the estimated cost-effectiveness of Wolbachia‑based biocontrol interventions. However, real-world implementation of Wolbachia-based biocontrol interventions is shaped by operational feasibility, community engagement, regulatory pathways, and health‑system capacity. These factors are as critical as economic evidence when considering the adoption of this intervention. Country ownership, the availability of the technology from different sources, and financing arrangements require careful consideration to ensure programs can be scaled and maintained in an equitable manner.*

*The equity implications surrounding Wolbachia‑based biocontrol interventions are also complex. On the one hand, significantly reducing the incidence of dengue can benefit poorer populations that face high* out-of-pocket*‑of costs associated with the disease. However, while targeting the intervention to ‑pocket costs associated with the disease. However, while targeting the intervention to high‑burden urban areas can maximise health gains and economic benefits, concentrating deployment in cities risks widening geographic and socioeconomic inequities if rural and peri-urban populations are left without access to disease control. This highlights that comprehensive dengue control in endemic countries will require multiple complementary interventions and should not rely on a single solution.*

**Summary and General Comments**
Reviewer #1: Dengue scoping review – General comments
In a spirit of transparency, this reviewer is willing to provide his identify to the authors: Donald Shepard.

This reviewer commends the authors for incorporating updated information where available. For example, the last sentence of the section “Effectiveness of the intervention” noted that Wolbachia had remained established in Australia for 15 years as of the time of the authors’ submission.

This reviewer also commends the inclusion of the burden of persistent dengue in economic evaluations. They note that some economic studies have included that component and appropriately recommend further research to improve understanding of the underlying variability.

As established researchers, the authors are undoubtedly aware of the varied phrasing that different authors use in reporting their findings. They may wish to reflect this understanding more fully in classifying which findings are “not reported.” For example, in the authors’ Table 3, the authors indicate that the ICER was not reported for reference 12 (Shepard et al). However, Shepard et al. report the net cost for their 11 target cities as negative $134,139.468, i.e., a negative number. In this case, an ICER cannot be calculated, Shepard et al similarly note Wolbachia is cost saving in locations where the incidence is at least 500 symptomatic cases per 100,000 population per year. The “National” (i.e. all cities) estimate is substantially above this threshold.

The authors’ entry in Table 3 for Shepard et al (ref 12 in manuscript and ref 16 in supplementary material) give a benefit-cost ratio of 4.68. Shepard et al. also report aggregate costs, so that aggregate benefits in millions of dollars can be calculated from the information provided. The authors may wish to reconsider the entry “not reported.”

Response: We fully agree regarding the varied phasing and the issue around negative ICERs. We have:

- Rechecked reference 12 and updated the results accordingly – accounting for additional information in the full paper mentioned as per the next comment. With this we have added 184.72 as the aggregate economic benefit in the supporting table.
- When the results relate to a negative ICER we have added cost-saving and removed the specific values.
- Changed our wording from “Not reported” to “Not explicitly reported” – to more clearly reflect that there are times where the numbers could be reconstructed

Similarly, while noting the cutoff dates for their review authors of scoping reviews sometimes incorporate updates or supplemental information. For example, the final version related to reference 12 was subsequently published as follows:
Shepard DS, Lee SR, Halasa-Rappel YA, Rincon Perez CW, Harker Roa A. Economic evaluation of Wolbachia deployment in Colombia: A modeling study. PLoS One 2025; 20(4): e0307045. <https://doi.org/10.1371/journal.pone.0307045>. Supporting information: [https://doi.org/10.1371/journal.pone.0307045.s001 through s006](https://doi.org/10.1371/journal.pone.0307045.s001 through%20s006)

Response: We have adapted the study to also refer to the published peer-reviewed version and added the following to the methods.

*In cases where a non peer–reviewed study was identified and a peer‑reviewed version was later published, we used the peer‑reviewed version, even if it appeared after the search timeframe.*

The manuscript uses the word “burden” 42 times. The authors’ text first uses the term “baseline burden” on p 6, where they note that they abstracted the “baseline burden of symptomatic cases.” As an area-based vector control technology, Wolbachia depends two measures of burden: dengue incidence per 100,000 population and population density (persons per kilometer). Shepard et al (ref 12, Fig 5B of published version) show how the combination of these two measures lead to a high “burden” and the most favorable benefit-cost ratios (the highest, 8.85 in the city of Bucaramanga, with the highest population density). This reviewer urges the authors to consider whether this two-dimensional definition of burden deserves highlighting for policy recommendations.

RESPONSE: We agree that this is important to highlight and have added the following into the discussion;

*It is important to consider that, being an area-based intervention, two aspects of burden need to be considered: the dengue incidence per 100,000 population and the population density of that area. Shepard et al. {Shepard, 2025 #413} demonstrated that the combination of these two aspects leads to a high burden area and the most favourable benefit-cost ratios, where from areas with the highest population density.*

**Areas that need further research**

Projecting the burden of dengue infection and disease

*…………………………. A range of measures can be used to characterise dengue burden, and different metrics will be appropriate when assessing the suitability of areas for different types of intervention. For an area‑based intervention, it is particularly important to consider both the incidence of infection and the population density of the target area, as these jointly determine the concentration of burden and the potential impact of control efforts.*

In comparing the replacement versus suppression Wolbachia strategies (p11), this reviewer encourages the authors to offer more policy insights about the results to date on this important question. For example, their discussion could suggest how the results on the suppression strategy from Singapore might be adapted to low- and middle-income countries to be compared with the replacement strategies. Similarly, in discussing the interplay between vaccination and Wolbachia, the cost structure of the two preventive interventions already generates important insights. Wolbachia costs depend primary on the square kilometers covered, whereas vaccination costs depend on the number of persons vaccinated. These patterns suggest that Wolbachia may be preferable in dense urban areas, whereas vaccination may be especially cost effective in areas of high dengue incidence but lower population density. The authors’ conclusions that “Further studies are needed…” (p11) and “There is a need to investigate…” (p 18) are always true and apply to every topic. This paper should maximize its contribution to policy as a careful and comprehensive review.

Response: These are important suggestions, and we have added the more focused policy insights surrounding the different cost structures

*We found seven studies evaluating Wolbachia-based interventions, six of which related to replacement-based strategy (mostly based on data related to the wMel Wolbachia strain). That said, there remains a need to evaluate Wolbachia replacement based programmes in other settings as well as the use of other the use of strains/distribution methods as they become available. There is also a need to further evaluate the cost-effectiveness of suppression-based strategies. It is also important to consider combinations of Wolbachia interventions. A key difference from both an operational and cost perspective is that suppression‑based Wolbachia strategies require ongoing releases, whereas replacement‑based strategies do not. This fundamental distinction has major implications for feasibility in LMIC settings. Implementing suppression at the same geographic scale as replacement would likely be substantially more costly. However, suppression approaches may still be suitable for targeted, localised hotspots where sustained releases can be focused on smaller areas with high transmission intensity. Further analysis is needed to understand how these methods perform operationally at different spatial scales, and what cost structures emerge under real‑world implementation conditions.*

*Further evaluation of the use of Wolbachia-based interventions in combination with other preventive interventions is needed. This is particularly important as new vaccines become available [74]. In this context, it is important to consider that although Wolbachia-based interventions can be highly cost-effective, it does not mean that it is possible to use them everywhere across endemic areas, particularly in more rural areas. The different cost structures of Wolbachia-based interventions and vaccination become highly relevant here. Wolbachia programme costs depend primarily on the geographic area that must be covered, whereas vaccination costs scale with the number of individuals vaccinated. These structural differences have notable implications for where each intervention is likely to be most cost-effective. There is a need to investigate the combination of Wolbachia-based interventions and vaccination more comprehensively, considering the use of different interventions in different areas within countries (rural vs. urban areas).*

Regarding making further direct policy-related conclusions on replacement vs suppression, we feel it is important not to overgeneralize/overreach based solely on the one study on suppression in Singapore. We therefore believe it is important to highlight that further studies are conducted on this topic before more policy recommendations are made on different Wolbachia strategies.

The variability in the effectiveness of Wolbachia is an important consideration, as the authors note on p15. However, a more balanced discussion would be helpful. The authors may also want to discuss experience from observational studies around Medellin, Colombia, summarized in the publication above by Shepard et al (2025). By avoiding some of the movement factors noted from Yogyakarta, Wolbachia proved more effective in Colombia.

Response: We have added the following content on the Colombia studies:

*“Several of the evaluations of the replacement-based strategy based the assumed effectiveness of Wolbachia deployments on the results of a cluster randomised trial and quasi-experimental studies in Yogyakarta (with the wMel strain) [28, 29]. There are at least three factors that can result in an underestimated efficacy from this type of trial: human movement, mosquito movement, and coupled transmission dynamics between trial arms [66]. Regarding the latter, Cavany et al. [66] highlighted the importance of transmission dynamic modelling in designing and interpreting future trials. A reanalysis of the Yogyakarta trial using spatiotemporally resolved data on the distribution of* Wolbachia *mosquitoes and the mobility of participants estimated an increased intervention efficacy to >80% [67]. In Colombia, following pilot releases in 2015–2016, staged city-wide Wolbachia deployments were undertaken in the cities of Bello, Medellín and Itagüí between October 2016 and April 2022 {Velez, 2023 #319}{Velez, 2023 #318}. A quasi-experimental study using interrupted time series analysis showed notified dengue case incidence in the three cities* declined by *95-97% compared to the prior decade {Velez, 2023 #318}.*

Reviewer #2: This manuscript presents a scoping review of economic evaluations on the cost-effectiveness of Wolbachia-based interventions, noting that the results indicate that this control method is cost-effective in large urban settings. This is a welcome discussion, especially considering the urgency of addressing rising cases of dengue and need for evidence-based policy regarding effective mosquito control methods. My main concern is the use strong language indicating a high degree of support for findings that are presented in a mostly qualitative and narrative manner, and based on a review of heterogeneous and small number of studies.
For example, the authors use the word ‘consensus’ in the abstract, author summary and discussion to indicate that ‘there is consensus that Wolbachia-based replacement interventions can be cost-effective for dengue control when targeted to high-burden settings’, but the evidence base is small, with heterogeneity in assumptions of baseline burdens as the authors themselves recognise. I would suggest rephrasing as ‘the available economic evaluations consistently suggest’, even more so considering that, to determine cost-effectiveness of the intervention, authors seem to be mostly comparing against a single numerical rule (0.5 threshold), for which there is some criticism, particularly regarding the need for such thresholds to reflect a country’s budget and context. However, this is not discussed as part of the assumptions made by the study, nor in areas that need further research section. I strongly recommend that authors justify this choice in more detail, with the appropriate interpretation caveats, as these studies have been conducted in countries with very different settings and especially because this manuscript could be of interest for decision-makers considering this control strategy.

Response: We fully agree with this point. In hindsight, consensus was a poor choice of words, and we have carefully edited the paper to ensure that this is fully removed. We have adopted the reviewer-suggested wording to “the available economic evaluations consistently suggest,”, which is what we intended to mean.

We have added further text on the choice of the 0.5 threshold – justifying it in the methods, noting that it is a debated area, and adding a clear corresponding limitation.

Methods: *To provide overarching, high-level conclusions, cost‑effectiveness ratios were compared against a threshold of 0.5 times a country’s per‑capita GDP [55, 58]. This benchmark is gaining traction as an alternative to the earlier (widely criticised) benchmarks that classified interventions as “cost‑effective” if they fell below 1–3 times per‑capita GDP per DALY averted [51]. However, it remains important to recognise that appropriate cost-effectiveness thresholds continue to be debated, and GDP‑based benchmarks have well-recognised limitations that should be considered when interpreting these findings [53, 54, 57, 59, 60, ]{Bertram, 2016 #320}.*

Limitations: *To provide overarching, high‑level conclusions, cost‑effectiveness ratios were compared against a threshold of 0.5 times a country’s per‑capita GDP. It is important to acknowledge that this type of standardised benchmark has notable limitations, particularly when applied across diverse settings. Ideally, policymakers should draw on locally appropriate thresholds that better reflect country‑specific opportunity costs, and/or budget constraints when making decisions.*

Another major comment pertains to mixing cost years and currencies without standardisation. Costs in Table 2 are reported in US$ but without specifying price years (this is sometimes specified in the manuscript’s text, but not for every estimate presented). Considering the wording of some results, such as ‘The assumed base case total cost per person covered within the evaluations of the replacement-based strategy varied between US$2.47-44.5 per person..’, I couldn’t determine if these values were standardised to a common price year, or underwent inflation adjustment. Ideally, authors would convert all costs to a common year and state this in methods.

Response: We have adjusted all of the cost values for inflation, adjusting to 2024 values

*Methods: Cost values (and economic benefits) were adjusted for inflation to 2024 prices using United States (US) gross domestic product (GDP) deflators [11]{Turner, 2019 #152}. ICERs values were not adjusted and reported with the corresponding cost year.*

The mostly narrative style of the manuscript and the table-format presentation means the reader must mentally integrate long textual descriptions and scan through large tables for each setting’s population size, cost per person and other important information to draw our own conclusions. I strongly suggest including some basic data visualization to improve clarity and support the manuscript conclusions. Perhaps a figure plotting cost per person protected vs. population size.

Response: We have added the following two figures:

| Populuation covered - millions | Cost per person covered |
| --- | --- |
| 0.46 | 15.4094 |
| 2.14 | 17.4236 |
| 11.19 | 14.463 |
| 2.44 | 25.735 |
| 20.02 | 10.163 |
| 0.225 | 14.6814 |
| 0.054 | 53.9937 |
| 22.12 | 2.62241 |
| 8.34 | 5.61579 |

**Figure 2: The reported cost per person covered compared to the population covered.** Labels next to each point indicate the population size for that setting. Only costs related to primary data were considered (hypothetical cost benchmarks/targets were not plotted). Uses the data related to the accelerated scenario for Brady *et al.* [25].

| Study setting | Societal benefit cost ratio (US$) |
| --- | --- |
| Port Vila, Vanuatu | 0.19 |
| Suva, Fiji | 1.67 |
| Yogyakarta City, Indonesia | 2.35 |
| Yogyakarta Special Autonomous Region, Indonesia | 1.44 |
| Bali, Indonesia | 1.35 |
| Jakarta, Indonesia | 3.4 |
| Vietnam (ten high burden cities) | 1.75 |
| Colombia (11 priority cities) | 4.68 |

**Figure 3: The projected societal benefit cost ratios across different study settings**. Bars show the estimated societal benefit–cost ratio for each implementation setting, reflecting the extent to which projected societal benefits outweigh programme costs. The dashed horizontal line at 1.0 indicates the break‑even point where benefits equal cost.

Reviewer #3: This is a relevant and original article as there does not exist a scoping revision of economic evaluations of Wolbachia-based interventions for dengue control, being for interest in the field of NTDS but also for researchers or practitioners outside the field. The manuscript requires significant revisions to improve its methodological rigor and analytical depth. This is a major limitation for a review comparing economic outcomes and undermines the reliability of its conclusions. To strengthen the work, the authors must (1) formally assess the quality of included studies using an appropriate tool and use this assessment to inform the interpretation of results.

RESPONSE: We have added quality assessment of the studies, as outlined in response to your comment in the Methods section of the comments

(2) The lack of a registered protocol should be explicitly justified in the text, and the study protocol should be provided as supplementary material.

Response We have added the following to the methods and will justify this to the editor.

*Because this scoping review was undertaken as an iterative exploratory exercise intended to map the emerging evidence, no protocol was registered at the time the review was initiated.*

Regarding the request to provide a study protocol as supplementary material, we respectfully disagree. As no protocol was developed, producing one retrospectively would not enhance transparency and would largely duplicate the methodological detail already provided in the manuscript. We have instead ensured that all relevant methodological steps are clearly and comprehensively described in the text.

#(3) The search strategy must be expanded to include a more systematic and documented coverage of grey literature (e.g., institutional reports, theses), detailing precisely what was searched and what was found.

Response: We have extended the search of the grey literature, as outlined in response to your comment in the Methods section of the comments

(4) The synthesis should move beyond listing results; where data permit, results should be standardized into comparable metrics like ICERs. Where direct comparison is not possible, the limitations must be clearly stated to avoid unqualified qualitative comparisons. Potential statistical graphs or analysis can be made from the data and it is worth to include them, and also to made accesible to the data in a repository. Finally, (5) the discussion must be expanded to include a critical analysis of how key drivers of heterogeneity—such as the assumed duration of effect and variation in effectiveness estimates—influence the conclusions, using concrete examples from the reviewed studies.

`Response: We have ensured that the relevant ICERS are summarized in a results Table and added more text in the results. The raw data is now available in a supporting file and we have added two new figures. Please note that significant methodological variation limits meaningful statistical analysis of the ICERs – which is why a meta-analysis was not conducted. Due to this significant variation (such as many studies reporting gross ratios rather than ICERs) graphical presentation was not found to be possible in a meaningful way. We have added the following as outlined in an earlier response:

*Results:*

*The cost-effectiveness ratios stratified by the different perspectives employed are summarised in Table 3 (with the corresponding economic benefits reported in Supporting Information file 1: Supporting Table S1). For this intervention, the broader the perspective, the lower the cost-effectiveness ratio. This is because the broader the perspective the higher the averted cost of illness associated with dengue cases; with the societal perspective including the estimated monetary value of the prevented productivity losses that would have been associated with a dengue case. For the same reason, the projections based on gross cost‑effectiveness ratios (ignoring all cost offsets) appeared less favourable then ICER based output. Differences in the chosen analytic perspective as well as the types of ratios reported, made further direct comparison across studies challenging. As a result, further quantitative synthesis of the cost‑effectiveness*

*Discussion:*

*There was notable variation in how results were reported across studies. This heterogeneity limited the scope for further quantitative analysis and prevented more direct comparison of the findings. To help address these challenges, Box 1 outlines recommendations to support future cost-effectiveness analysis of Wolbachia-based biocontrol interventions.*

**Additional Editor Comments:**

The reviewers agree that this is a timely and relevant scoping review addressing an important policy question, and commend the authors for compiling and updating a dispersed literature on the cost-effectiveness of Wolbachia-based dengue control. However, substantial revisions are required to improve methodological rigor, analytical depth, clarity, and policy relevance before the manuscript can be reconsidered. Please make sure you thoroughly address the points I summarized below.

1. Strengthen methodological tansparency and rigor by
• substantially revising the Methods and limitations to address concerns about review conduct and reliability
• explicitly justifying the lack of a registered protocol (e.g., PROSPERO), and provide the study protocol as supplementary material if available.
• clearly describing procedures used to minimize bias (e.g., independent screening, dual data extraction, consensus processes), particularly in light of disclosed conflicts of interest.
• adding a formal quality / risk-of-bias assessment of included economic evaluations using an appropriate tool, and use this assessment to inform interpretation and conclusions.

2. Improve Consistency, Standardization, and Reporting of Economic Data:
• cost standardization: clearly state price years for all cost estimates; where possible, convert costs to a common currency and base year, and explain inflation or exchange rate adjustments.
• avoid mixing currencies and price years without explicit standardization.

3. Move beyond narrative listing to analytical synthesis
All reviewers noted that the manuscript relies too heavily on descriptive narration:
• dd basic quantitative synthesis and visualization where data permit (e.g., plots of cost per person protected vs. population size or density).
• Where standardization is possible (e.g., ICERs, cost per person covered), present results in comparable metrics.
• Where comparison is not possible, explicitly state why, rather than drawing qualitative conclusions.
• Consider making extracted data available in a repository.

4. Address key drivers of heterogeneity and interpretation
The Discussion must more critically analyze why estimates differ across studies and explicitly examine how differences in:
• baseline burden definitions, population density, effectiveness assumptions and duration, disease measurement and disability weights, inclusion/exclusion of vector control costs,time horizons, affect cost-effectiveness conclusions.

• Consider highlighting burden as a multidimensional concept (e.g., incidence × population density) and its implications for policy targeting.

5. Tone down over-strong language and justify assumptions
• Rephrase claims suggesting “consensus” to reflect the small, heterogeneous evidence base (e.g., “available studies consistently suggest…”).
• Justify the use of cost-effectiveness thresholds (e.g., 0.5 GDP-based rules), discuss their limitations, and interpret results in light of country-specific budget contexts.

6. Expand policy, equity, and implementation discussion
Discuss implications of centralized control and accessibility of Wolbachia technology, including issues of transparency, national capacity, and long-term sustainability.

7. Deepen comparative and forward-looking policy insights
• Provide more substantive policy discussion comparing replacement vs. suppression strategies, Wolbachia vs. vaccination, considering their fundamentally different cost structures (area-based vs. person-based).
• Avoid generic conclusions (“further studies are needed”) and instead articulate specific, decision-relevant insights.

8. Technical and structural revisions
• Relocate operational distinctions (e.g., replacement vs. suppression logistics) to the Methods where appropriate.
• Define key categorizations clearly (e.g., “small-scale” vs. large urban settings) and make them explicit in tables
• Correct typographical errors and missing verbs noted by reviewers.

Response: Many thanks for highlighting these comments. These have been carefully considered and in light of our revisions to the reviewers comments

**Journal Requirements:**

If the reviewer comments include a recommendation to cite specific previously published works, please review and evaluate these publications to determine whether they are relevant and should be cited. There is no requirement to cite these works unless the editor has indicated otherwise.

1) Please ensure that the CRediT author contributions listed for every co-author are completed accurately and in full.
At this stage, the following Authors/Authors require contributions: Hugo C Turner, Trinh Manh Hung, Oliver J Brady, Raman Velayudhan, Ilaria Dorigatti, and Hannah E Clapham. Please ensure that the full contributions of each author are acknowledged in the "Add/Edit/Remove Authors" section of our submission form.
The list of CRediT author contributions may be found here: <https://journals.plos.org/plosntds/s/authorship#loc-author-contributions>

Response: We have added CRediT author contributions

2) We do not publish any copyright or trademark symbols that usually accompany proprietary names, eg ©,  ®, or TM  (e.g. next to drug or reagent names). Therefore please remove all instances of trademark/copyright symbols throughout the text, including:
- ® on page: 13.

Response: We have removed the ®

3) We have noticed that you have uploaded Supporting Information files, but you have not included a list of legends. Please add a full list of legends for your Supporting Information files after the references list.

Response: We have added a full list of legends for our Supporting Information files.

4) Some material included in your submission may be copyrighted. According to PLOSu2019s copyright policy, authors who use figures or other material (e.g., graphics, clipart, maps) from another author or copyright holder must demonstrate or obtain permission to publish this material under the Creative Commons Attribution 4.0 International (CC BY 4.0) License used by PLOS journals. Please closely review the details of PLOSu2019s copyright requirements here: PLOS Licenses and Copyright. If you need to request permissions from a copyright holder, you may use PLOS's Copyright Content Permission form.
Please respond directly to this email and provide any known details concerning your material's license terms and permissions required for reuse, even if you have not yet obtained copyright permissions or are unsure of your material's copyright compatibility. Once you have responded and addressed all other outstanding technical requirements, you may resubmit your manuscript within Editorial Manager. 
Potential Copyright Issues:
i) Figure 1. Please confirm whether you drew the images / clip-art within the figure panels by hand. If you did not draw the images, please provide (a) a link to the source of the images or icons and their license / terms of use; or (b) written permission from the copyright holder to publish the images or icons under our CC BY 4.0 license. Alternatively, you may replace the images with open source alternatives. See these open source resources you may use to replace images / clip-art:
- [https://commons.wikimedia.org](https://commons.wikimedia.org/)
- <https://openclipart.org/>.

Response: The mosquito cartoon image we used was open source but it appears that the original image has been removed. Due to this, we have removed Figure 1 – adding additional figures to the results instead.

5) We note that your Data Availability Statement is currently as follows: "All data analysed or generated during this study are included in this article and its additional material". Please confirm at this time whether or not your submission contains all raw data required to replicate the results of your study. Authors must share the “minimal data set” for their submission. PLOS defines the minimal data set to consist of the data required to replicate all study findings reported in the article, as well as related metadata and methods (<https://journals.plos.org/plosone/s/data-availability#loc-minimal-data-set-definition>).
For example, authors should submit the following data: 
1) The values behind the means, standard deviations and other measures reported;
2) The values used to build graphs;
3) The points extracted from images for analysis..
Authors do not need to submit their entire data set if only a portion of the data was used in the reported study.
If your submission does not contain these data, please either upload them as Supporting Information files or deposit them to a stable, public repository and provide us with the relevant URLs, DOIs, or accession numbers. For a list of recommended repositories, please see <https://journals.plos.org/plosone/s/recommended-repositories>. 
If there are ethical or legal restrictions on sharing a de-identified data set, please explain them in detail (e.g., data contain potentially sensitive information, data are owned by a third-party organization, etc.) and who has imposed them (e.g., an ethics committee). Please also provide contact information for a data access committee, ethics committee, or other institutional body to which data requests may be sent. If data are owned by a third party, please indicate how others may request data access.

Response: All of the values were reported in the first submission in the main tables. In light of the changes with have made we have added a full Excel file with all the raw data and the values adjusted for inflation. This has been added as a supporting file.

6) Please amend your detailed Financial Disclosure statement. This is published with the article. It must therefore be completed in full sentences and contain the exact wording you wish to be published.
1) State the initials, alongside each funding source, of each author to receive each grant. For example: "This work was supported by the National Institutes of Health (####### to AM; ###### to CJ) and the National Science Foundation (###### to AM)."
2) State what role the funders took in the study. If the funders had no role in your study, please state: "The funders had no role in study design, data collection and analysis, decision to publish, or preparation of the manuscript."
3) If any authors received a salary from any of your funders, please state which authors and which funders..
If you did not receive any funding for this study, please simply state: u201cThe authors received no specific funding for this work.u201d

Response: We have edited the statement accordingly.

7)  Please ensure that the funders and grant numbers match between the Financial Disclosure field and the Funding Information tab in your submission form. Note that the funders must be provided in the same order in both places as well.

Response: Will have ensured this during the resubmission.
