# Supplementary material for: The cost-effectiveness of Wolbachia-based biocontrol interventions for dengue: A scoping review of the available evidence
Source: PLoS Negl Trop Dis. 2026 Jun 1;20(6):e0014395. doi: 10.1371/journal.pntd.0014395 (PMC13245869; doi:10.1371/journal.pntd.0014395)
Supplement: Attachment — Submitted filename: Wol health econ Comments round 2_hc.docx [file pntd.0014395.s005.docx]

The manuscript is well-executed, clearly written, and makes a valuable and policy-relevant contribution to the field. The reviewers are broadly supportive, and the requested at this stage revisions are Minor and primarily focused on clarity, consistency, and strengthening the alignment between results and interpretation. Please refer to these priority revisions for final acceptance:
1) Strengthen the abstract and key messaging:
• Make the main findings more explicit and informative (particularly cost-saving implications), and replace vague or generic concluding statements with specific takeaways.
• Align conclusions with the evidence presented.
• Ensure the discussion accurately reflects the strength of the findings, particularly regarding the consistent cost-effectiveness of replacement strategies, and avoid unnecessary calls for further research where evidence is already clear.

Response: We thank the reviewers and editor for their constructive feedback. In response, we have strengthened the key messaging in the abstract, author summary, and conclusions—particularly by making the cost‑saving implications more explicit and ensuring that the conclusions clearly reflect the consistent evidence supporting the cost‑effectiveness of Wolbachia replacement strategies in densely populated urban settings. We have also revised the reference to making recommendations for future analyses to be more specific and action‑oriented and less vague.

Regarding the comment on avoiding unnecessary calls for further research, we fully agree that the evidence for replacement strategies is strong and have ensured that this is more clearly conveyed. However, we consider it important to retain a brief statement highlighting remaining uncertainties related to heterogeneity in epidemiological effectiveness and how the need to look at the investigate the combination of Wolbachia-based interventions with other dengue control/prevention measures in the abstract. As this scoping review aims to identify both the strengths of current evidence and gaps in the literature, we believe this focused statement appropriately reflects the current state of knowledge without undermining the robustness of findings for replacement programmes.

*Abstract: Conclusions/Significance.*

*The available economic evaluations consistently suggest that Wolbachia-based replacement interventions can be cost-effective for dengue control when targeted to densely populated urban areas, and several studies indicate that they can generate substantial long-term cost savings from a societal perspective. Further research is needed to understand how heterogeneity in epidemiological effectiveness influences long-term projected cost-effectiveness and to investigate the combination of Wolbachia-based interventions with other dengue control/prevention measures (such as vaccination). To support more robust and comparable analyses, we provide recommendations for future studies in this area, emphasising the importance of reporting results disaggregated by cost and outcome components, and making underlying assumptions related to the intervention more explicit.*

*Author Summary*

*Dengue poses a growing global health threat, with nearly half the world’s population at risk. Among emerging control strategies, Wolbachia-infected mosquitoes offer a promising biocontrol approach—either by replacing local mosquito populations with less infectious ones or by suppressing mosquito numbers. We conducted the first scoping review of economic evaluations of Wolbachia-based interventions for dengue control, identifying nine studies published up to 29 April 2024. Most focused on replacement strategies, particularly in large urban settings, and found these interventions to be cost-effective from a societal perspective. Our review highlights that the available economic evaluations consistently suggest that Wolbachia‑based replacement programmes are highly cost‑effective in high‑burden areas, where the intervention has the potential to generate substantial long‑term cost savings from a societal perspective in many settings. We also identify key drivers of variation across studies and provide recommendations to guide future economic evaluations in this field, emphasising the importance of reporting results in a disaggregated manner and making relevant underlying assumptions related to this intervention more explicit.*

We are also streamlining the main conclusion section to ensure it is aligned with the evidence presented. Note that our intention is to ensure that the evidence base is not overstated, which is why we are highlighting that the heterogeneity in impact and variation in long-term effectiveness needs to be considered across a greater range of wider range of operational and epidemiological settings. Without this, the finding could more easily be taken out of context. Further justification for including this statement is outlined in our response to the reviewers.

***Conclusion***

*Based on the currently available evidence, there is consensus that Wolbachia-based replacement interventions can be cost-effective for dengue control, when they are appropriately targeted, particularly in high-density urban environments (such as cities). For such high burden settings, the estimated net cost-effectiveness ratios in terms of the cost per DALY averted from the health sector perspective were typically below 0.5 the per capita GDP cost-effectiveness threshold. When taking the societal perspective and including the monetary value of the productivity gains associated with the averted cases, the economic benefits of a Wolbachia-based replacement intervention often outweigh its cost. A suppression-based strategy was also found to be cost-saving in Singapore from a societal perspective, however it had a notably higher cost per person covered compared to the replacement-based interventions.*

*That said, in settings where the baseline burden is not sufficiently high, this type of intervention is unlikely to be cost-effective. The exact conditions which determine which areas should be targeted will be setting specific. That said, in settings where the baseline burden is not sufficiently high, this type of intervention is unlikely to be cost-effective. The exact conditions which determine which areas should be targeted will be setting specific. It is important to note that the successful dispersal of Wolbachia-infected mosquitoes can be heterogeneous and influenced by local environmental factors. Further studies are needed to investigate and quantify how this heterogeneity affects long-term projections of the cost-effectiveness of Wolbachia-based interventions across a greater range of wider range of operational and epidemiological settings. ……*

2) Enhance clarity, transparency, and consistency:
• Address minor issues in wording precision, referencing (including use of final published sources), and table reporting (e.g., clearer terminology and inclusion of simple derived metrics where appropriate).

Response: We have addressed the points – see responses to the Reviewer.

**Journal Requirements:**

If the reviewer comments include a recommendation to cite specific previously published works, please review and evaluate these publications to determine whether they are relevant and should be cited. There is no requirement to cite these works unless the editor has indicated otherwise

**Reviewers' comments:**

Reviewer's Responses to Questions

**Key Review Criteria Required for Acceptance?**


As you describe the new analyses required for acceptance, please consider the following:


**Methods**

-Are the objectives of the study clearly articulated with a clear testable hypothesis stated?

-Is the study design appropriate to address the stated objectives?

-Is the population clearly described and appropriate for the hypothesis being tested?

-Is the sample size sufficient to ensure adequate power to address the hypothesis being tested?

-Were correct statistical analysis used to support conclusions?

-Are there concerns about ethical or regulatory requirements being met?

Reviewer #1: Yes. The methods of this scoping review are appropriate. This reviewer commends the many improvements reflected in this revision.

Reviewer #3: Yes

**Results**


-Does the analysis presented match the analysis plan?

-Are the results clearly and completely presented?

-Are the figures (Tables, Images) of sufficient quality for clarity?

Reviewer #1: Results only partially match the analysis plan. The very different results between the replacement and suppression strategies needs further highlighting. As authors who seek to inform policy, they should make maximum use of information which is indirectly reported to estimate the long term cost-effectiveness of the suppression strategy and compare it to the replacement strategy.

Response: We have added further results on the suppression strategy. They did also find it to be cost-saving from a societal perspective (over the 11 years investigated), and we have added these newly estimated BCRs into the paper, and highlighted this result.

The main difference in the results is that the cost per person covered is substantially higher for Wolbachia‑based suppression strategies than for replacement strategies. However, the evidence base is currently too limited to support firm conclusions beyond this. With only a single study evaluating suppression, it is not possible to reliably disentangle how much of the observed difference reflects intrinsic differences between intervention types versus higher implementation and operational costs associated with implementing in Singapore. We have added more text to highlight relevant results between replacement and suppression strategies. However, we believe it is inappropriate to draw too many firm conclusions based on only one study. This is the reason we are calling for more research.

We have added the following to the results:

*When using the societal perspective, a national suppression-based strategy in Singapore was also projected to generate cost-savings. with societal benefit-cost ratios >1[34] (Table 3 and Supporting Information file 1: Supporting Table S1). However, it had a notably higher cost per person covered (US$59 per person covered - Table 2) over the 11 years of the intervention compared to the replacement-based interventions at a similar scale (Figure 2).*

Reviewer #3: Yes

**Conclusions**


-Are the conclusions supported by the data presented?

-Are the limitations of analysis clearly described?

-Do the authors discuss how these data can be helpful to advance our understanding of the topic under study?

-Is public health relevance addressed?

Reviewer #1: The revision is improved over the previous version. Nevertheless, the comment from results applies to the conclusions and is repeated here:

The very different results between the replacement and suppression strategies needs further highlighting. As authors who seek to inform policy, they should make maximum use of information which is indirectly reported to estimate the long term cost-effectiveness of the suppression strategy and compare it to the replacement strategy.

Response -We have added the following into the conclusion.

*A suppression-based strategy was also found to be cost-saving in Singapore from a societal perspective, however it had a notably higher cost per person covered compared to the replacement-based strategy.*

Reviewer #3: Yes

**Editorial and Data Presentation Modifications?**


Use this section for editorial suggestions as well as relatively minor modifications of existing data that would enhance clarity. If the only modifications needed are minor and/or editorial, you may wish to recommend “Minor Revision” or “Accept”.

Reviewer #1: Note: This reviewer used the page numbers in the electronic pdf version, which includes four background pages before the revised draft manuscript. Thus, PDF p 5 is the title page (p 1 as labeled by the authors).

Pdf P 1 The following phase in the abstract is currently in parentheses: (with the intervention likely to generate cost savings from a societal perspective). Given the importance of this finding, it should not be enclosed in parentheses, but instead separated by comma.

Response: We agree and have made that change

Pdf P 1 (abstract) and P 7 (authors’ summary). As the authors know, most readers seek informative abstracts. Most of the abstract meets this expectation, but the last sentence, which follows, falls short: “We outline recommendations for future economic evaluations in this area.” This reviewer recommends that that sentence, be deleted and the previous sentence be extended by beginning with the word “including” and inserting the most important recommendation.

Response We have expanded the sentence to include the most important recommendation. We respectfully disagree with the reviewer’s suggestion to delete the final sentence and merge its content into the preceding one. In our view, the recommendations we provide constitute a distinct and important contribution of the paper, and retaining a separate sentence helps ensure that this contribution is clearly signposted to readers. Combining it with the previous sentence would risk obscuring the broader methodological guidance we aim to highlight. We have therefore kept the sentence in place while ensuring that the abstract remains concise and informative.

Pdf P 2, Instead of “to date,” this reviewer suggests inserting the date of the authors’ cutoff date (29 April 2024).

Response: We have made the suggested change.

Pdf p 10. To avoid ambiguity, this reviewer suggests changing “Greater than 200,000…” to “At least 200,000…”

Response: We have made the suggested change.

PDF p 10. A more recent publication strengthens the authors’ choice of a threshold of 0.5 GDP/capita (currently based on refs 15 and 16). This reviewer suggests that the authors include a statement along the following lines: “An empirical analysis of historical spending across 174 countries found that that 51% implicitly used thresholds of cost per QALY of 0.5 times their per capita GDP (Pichon-Riviere et al., 2023).”

Response: We agree and have added the suggested text and reference.

Pichon-Riviere A, Drummond M, Palacios A, Garcia-Marti S, Augustovski F. Determining the efficiency path to universal health coverage: cost-effectiveness thresholds for 174 countries based on growth in life expectancy and health expenditures. Lancet Glob Health. 2023;11(6):e833-e42. doi:10.1016/S2214-109X(23)00162-6.

Pdf p 11. This reviewer agrees with the authors’ decision to include subsequent published versions of papers that had not been peer reviewed at the time of the authors’ search. As the authors indicated that they used the published version for their search, their citations in the text and tables should be broadened to include the published version in all places. For example, pdf p 11 refers only to the preprint version from Colombia (ref 25) but not the published version (ref 47).

Response: We have both references throughout the text when referring to that study.

Pdf p 16. This reviewer agrees only in part with the authors’ conclusion that Wolbachia studies have not generally addressed distributional elements. However, it would be helpful for the authors to add that Wolbachia is an area-based intervention that does not require any action from a resident to benefit. Therefore, it is likely to benefit all residents in the target area similarly. Accordingly, the authors could draw on an interview study across eight endemic countries in both Asia and the Americas (Suaya JA et al. Am. J. Trop. Med. Hyg., 80(5), 2009, pp. 846–855). In that study, 83% of the participants were urban, similar to the potential populations served by Wolbachia. That study found that 29% of respondents had primary education or less, 44% secondary, and 27% vocational or higher. Thus, it is likely the equity considerations of Wolbachia would be broad, benefiting residents across all socio-economic levels.

Response: We fully agree that, as an area‑based intervention that does not require any action from residents to benefit, Wolbachia is likely to provide broadly equitable protection for all individuals living within the release zone, including across socio‑economic groups. We have now highlighted this point in the revised text. At the same time, we consider it important to reflect the broader equity implications of the implementation strategy. If countries prioritise Wolbachia deployment primarily in large urban centres—where feasibility and population density are highest—this has distributional implications at the national level. Specifically, it may widen disparities in protection between urban and rural populations, where dengue burden can also be substantial but where Wolbachia deployment may be less feasible. We have therefore expanded the text on the discussion on this area.

*Implementation and equity*

*This review focused on the estimated cost-effectiveness of Wolbachia‑based biocontrol interventions. However, real-world implementation of Wolbachia-based biocontrol interventions is shaped by operational feasibility, community engagement, regulatory pathways, and health‑system capacity. These factors are as critical as economic evidence when considering the adoption of this intervention. Country ownership, the availability of the technology from different sources, and financing arrangements require careful consideration to ensure programs can be scaled and maintained in an equitable manner.*

*The equity implications surrounding Wolbachia‑based biocontrol interventions are also complex. On the one hand, significantly reducing the incidence of dengue can benefit poorer populations that face high out‑of‑pocket costs associated with the disease. In addition, as an area‑based intervention that does not require any action from residents to benefit, Wolbachia-based interventions are expected to provide broadly equitable protection for all individuals living within the release zone, including across socio‑economic groups. However, while targeting the intervention to high‑burden urban areas can maximise health gains and economic benefits, concentrating deployment in cities risks widening geographic and socioeconomic inequities if rural and peri-urban populations are left without access to disease control. This highlights that comprehensive dengue control in endemic countries will require multiple complementary interventions and should not rely on a single solution.*

Pdf p 17. The authors’ final result states that further studies are needed concerning the relative cost-effectiveness of replacement versus suppression. That statement is not consistent with the results of their scoping review, which finds that replacement is consistently cost-effective, and sometimes cost-saving. Furthermore, the authors should comment on Singapore’s previous suppression strategy through the country’s vigorous through intensive efforts to monitor and eliminate breeding sites through inspections and penalties. These policies had the perverse effect of lowering population immunity, contributing to subsequent large outbreaks. Furthermore, the authors noted that most suppression strategies assume a 10-year period of effectiveness. This, if program costs are annualized, the suppression approach is about ten times more costly than the replacement approach. If the calculations recommended below for pdf pp 38-41 are completed, I expect that the suppression approach will not be cost saving even though Singapore is a dense city. This paper would best serve the goal of informing policy through reasonable projections, with caveats, instead of the cliché of calling for more research.

Response: Regarding adding more contexts on Singapore’s we have added the following.

*At this time, there are limited data related to the effectiveness of suppression-based strategies, with the only evaluation assuming a wide range of effectiveness between 40-80%. There is a need for further effectiveness data, particularly outside of Singapore. It is important to note that historically, Singapore has implemented one of the world’s most intensive vector control programmes, with sustained efforts that drove Aedes populations to levels [72]. While initially highly effective in suppressing transmission and dengue incidence, this also resulted in a progressively larger pool of susceptible individuals, which has been identified as a key driver of the scale of subsequent outbreaks when new serotypes or substantial viral introductions occur and requiring increasing amounts of control measures to keep transmission at the same level [72][73]. These epidemiological implications of these dynamics [73] warrant greater consideration when evaluating the projected long-term impact of Wolbachia-based interventions.*

As outlined in other responses, we have done further calculations based on the information in the paper. As they reported yearly cost-savings we were able to reconstruct the cost-benefit ratios. Importantly, it is cost-saving from a societal perspective over the 11 years investigated. We have added more on this into the text and have highlighted more that suppression-based programmes will be more expensive.

Respectfully, we disagree that identifying areas for further research is a cliché; articulating research needs is a core purpose of scoping reviews. Our statement was intended to refer specifically to the relative cost-effectiveness of replacement versus suppression. While our scoping review finds that replacement is consistently cost effective—and in several cases cost-saving—the evidence base for suppression is more limited, with only a single economic evaluation available. It is also important that a wider range of operational and epidemiological settings are investigated. As a result, we do not believe it is possible to draw comparative conclusions between the two strategies at this stage – other than saying that suppression will be more expensive. Our wording is intended to reflect this asymmetry in the evidence rather than to imply uncertainty about the cost-effectiveness of replacement itself. We have changed the wording of the section highlighted by the reviewer to make this point clearer:

*We identified nine studies evaluating Wolbachia-based interventions for dengue control. The majority (eight out of nine studies) investigated Wolbachia replacement-based programmes. These available economic evaluations consistently suggest that Wolbachia-based replacement interventions can be cost-effective for dengue control when appropriately targeted, particularly in high-density urban environments such as cities. In contrast, only one study has evaluated a suppression‑based intervention, making it difficult to draw robust conclusions about its relative cost‑effectiveness. Although the data available indicate that suppression interventions will be more costly, further research is needed to enable more informative comparisons and to examine how the cost‑effectiveness of both approaches varies across a wider range of operational and epidemiological settings.*

We have edited the conclusion to include:

*A suppression-based strategy was also found to be cost-saving in Singapore from a societal perspective, however it had a notably higher cost per person covered compared to the replacement-based interventions.*

Pdf p 18. The authors wrote: The cost-effectiveness ratios from a societal perspective were often negative….” To be precise, the authors should consider inserting “of the replacement strategy….”

Response: Very good point and we have edited accordingly.

PDF P 27. Following recommended style, the authors have generally italicized the word Wolbachia. They should check their manuscript and improve consistency for places like the beginning of the second full paragraph on this page, where they did not italicize “Wolbachia-based.”

Response: We have checked this and made it consistent.

Pdf p 38-41. In Tables 2, the term “Not explicitly reported” is misleading. It would be more accurate to state “Indirectly reported.” By indicating that the program is national, the authors provide the data for deriving the underlying population, the cost per person, and benefit cost ratios. With the paper’s goal of informing the field, these simple calculations should be done to fill in the missing items, with explanations provided in footnotes or supplements.

Response: We have changed the term to “Not directly reported” when an item is not explicitly stated in the study, and we have filled in several of the previously missing items where possible. We believe this terminology is clear and accurately reflects cases where information is absent from the published report. We do not use the term “Indirectly reported”, as this would imply that the information can always be inferred, which is not consistently true across studies. It is also important to note that even when a programme is described as national, this does not necessarily mean that the intervention must target the entire geographical area.

| **Study** | **Setting** | **Baseline burden of symptomatic cases in the absence of the intervention** | **Base case – symptomatic cases per 100,000 per year** | **Base case effectiveness (percentage reduction in dengue cases)^1^** | **Base case duration of effectiveness assumed for base case results** | **Release area (Km^2^) covered** | **Population covered - millions** | **Scale** | **Base case intervention cost per person (US$ 2024)** | **Base case total intervention cost - US$ millions (2024 prices)** | **Original cost year** |
| --- | --- | --- | --- | --- | --- | --- | --- | --- | --- | --- | --- |
| Soh *et al.* 2021 | Singapore | Varied year by year between 2,767-35,315 cases | - | 40-80% | Not applicable | Not directly reported | Not directly reported: inferred to be 5,076,732 based on the population of Singapore in 2010 [13] | Large | Not directly reported: estimated to be approximately US$59^4^ | Not directly reported: estimated to be 297.34 over 11 years^5^ | 2010 |

*^4^* *Based on the assumed population size of Singapore and the estimated total cost of the intervention. ^5^Based on the reported yearly value of US$31.24* million *(discounted at 3% per year).*

Reviewer #3: Accept
