# Supplementary material for: The cost-effectiveness of Wolbachia-based biocontrol interventions for dengue: A scoping review of the available evidence
Source: PLoS Negl Trop Dis. 2026 Jun 1;20(6):e0014395. doi: 10.1371/journal.pntd.0014395 (PMC13245869; doi:10.1371/journal.pntd.0014395)
Supplement: Peer review history

# Peer review: Decision Letter 0

**DOI:** 10.1371/journal.pntd.0014395.r001

**Authors:** Nigel Beebe, Gordana Rasic

22 Dec 2025

PNTD-D-25-01795

The cost-effectiveness of Wolbachia-based biocontrol interventions for dengue: A scoping review of the available evidence

PLOS Neglected Tropical Diseases

Dear Dr. Turner,

Thank you for submitting your manuscript to PLOS Neglected Tropical Diseases. After careful consideration, we feel that it has merit but does not fully meet PLOS Neglected Tropical Diseases's publication criteria as it currently stands. Therefore, we invite you to submit a revised version of the manuscript that addresses the points raised during the review process.

Please submit your revised manuscript within by Feb 20 2026 11:59PM. If you will need more time than this to complete your revisions, please reply to this message or contact the journal office at plosntds@plos.org. When you're ready to submit your revision, log on to https://www.editorialmanager.com/pntd/ and select the 'Submissions Needing Revision' folder to locate your manuscript file.

Please include the following items when submitting your revised manuscript:

* A letter that responds to each point raised by the editor and reviewer(s). You should upload this letter as a separate file labeled 'Response to Reviewers'. This file does not need to include responses to any formatting updates and technical items listed in the 'Journal Requirements' section below.

* A marked-up copy of your manuscript that highlights changes made to the original version. You should upload this as a separate file labeled 'Revised Manuscript with Track Changes'.

* An unmarked version of your revised paper without tracked changes. You should upload this as a separate file labeled 'Manuscript'.

If you would like to make changes to your financial disclosure, competing interests statement, or data availability statement, please make these updates within the submission form at the time of resubmission. Guidelines for resubmitting your figure files are available below the reviewer comments at the end of this letter.

We look forward to receiving your revised manuscript.

Kind regards,

Gordana Rasic, Ph.D.

Academic Editor

PLOS Neglected Tropical Diseases

Nigel Beebe

Section Editor

PLOS Neglected Tropical Diseases

Shaden Kamhawi

co-Editor-in-Chief

PLOS Neglected Tropical Diseases

orcid.org/0000-0003-4304-636XX

Paul Brindley

co-Editor-in-Chief

PLOS Neglected Tropical Diseases

orcid.org/0000-0003-1765-0002

**Additional Editor Comments:**

The reviewers agree that this is a timely and relevant scoping review addressing an important policy question, and commend the authors for compiling and updating a dispersed literature on the cost-effectiveness of Wolbachia-based dengue control. However, substantial revisions are required to improve methodological rigor, analytical depth, clarity, and policy relevance before the manuscript can be reconsidered. Please make sure you thoroughly address the points I summarized below.

1. Strengthen methodological tansparency and rigor by

• substantially revising the Methods and limitations to address concerns about review conduct and reliability

• explicitly justifying the lack of a registered protocol (e.g., PROSPERO), and provide the study protocol as supplementary material if available.

• clearly describing procedures used to minimize bias (e.g., independent screening, dual data extraction, consensus processes), particularly in light of disclosed conflicts of interest.

• adding a formal quality / risk-of-bias assessment of included economic evaluations using an appropriate tool, and use this assessment to inform interpretation and conclusions.

2. Improve Consistency, Standardization, and Reporting of Economic Data:

• cost standardization: clearly state price years for all cost estimates; where possible, convert costs to a common currency and base year, and explain inflation or exchange rate adjustments.

• avoid mixing currencies and price years without explicit standardization.

3. Move beyond narrative listing to analytical synthesis

All reviewers noted that the manuscript relies too heavily on descriptive narration:

• dd basic quantitative synthesis and visualization where data permit (e.g., plots of cost per person protected vs. population size or density).

• Where standardization is possible (e.g., ICERs, cost per person covered), present results in comparable metrics.

• Where comparison is not possible, explicitly state why, rather than drawing qualitative conclusions.

• Consider making extracted data available in a repository.

4. Address key drivers of heterogeneity and interpretation

The Discussion must more critically analyze why estimates differ across studies and explicitly examine how differences in:

• baseline burden definitions, population density, effectiveness assumptions and duration, disease measurement and disability weights, inclusion/exclusion of vector control costs,time horizons, affect cost-effectiveness conclusions.

• Consider highlighting burden as a multidimensional concept (e.g., incidence × population density) and its implications for policy targeting.

5. Tone down over-strong language and justify assumptions

• Rephrase claims suggesting “consensus” to reflect the small, heterogeneous evidence base (e.g., “available studies consistently suggest…”).

• Justify the use of cost-effectiveness thresholds (e.g., 0.5 GDP-based rules), discuss their limitations, and interpret results in light of country-specific budget contexts.

6. Expand policy, equity, and implementation discussion

Discuss implications of centralized control and accessibility of Wolbachia technology, including issues of transparency, national capacity, and long-term sustainability.

7. Deepen comparative and forward-looking policy insights

• Provide more substantive policy discussion comparing replacement vs. suppression strategies, Wolbachia vs. vaccination, considering their fundamentally different cost structures (area-based vs. person-based).

• Avoid generic conclusions (“further studies are needed”) and instead articulate specific, decision-relevant insights.

8. Technical and structural revisions

• Relocate operational distinctions (e.g., replacement vs. suppression logistics) to the Methods where appropriate.

• Define key categorizations clearly (e.g., “small-scale” vs. large urban settings) and make them explicit in tables

• Correct typographical errors and missing verbs noted by reviewers.

**Journal Requirements:**

If the reviewer comments include a recommendation to cite specific previously published works, please review and evaluate these publications to determine whether they are relevant and should be cited. There is no requirement to cite these works unless the editor has indicated otherwise.

1) Please ensure that the CRediT author contributions listed for every co-author are completed accurately and in full.

At this stage, the following Authors/Authors require contributions: Hugo C Turner, Trinh Manh Hung, Oliver J Brady, Raman Velayudhan, Ilaria Dorigatti, and Hannah E Clapham. Please ensure that the full contributions of each author are acknowledged in the "Add/Edit/Remove Authors" section of our submission form.

The list of CRediT author contributions may be found here: https://journals.plos.org/plosntds/s/authorship#loc-author-contributions

2) We do not publish any copyright or trademark symbols that usually accompany proprietary names, eg ©,  ®, or TM  (e.g. next to drug or reagent names). Therefore please remove all instances of trademark/copyright symbols throughout the text, including:

- ® on page: 13.

3) We have noticed that you have uploaded Supporting Information files, but you have not included a list of legends. Please add a full list of legends for your Supporting Information files after the references list.

4) Some material included in your submission may be copyrighted. According to PLOSu2019s copyright policy, authors who use figures or other material (e.g., graphics, clipart, maps) from another author or copyright holder must demonstrate or obtain permission to publish this material under the Creative Commons Attribution 4.0 International (CC BY 4.0) License used by PLOS journals. Please closely review the details of PLOSu2019s copyright requirements here: PLOS Licenses and Copyright. If you need to request permissions from a copyright holder, you may use PLOS's Copyright Content Permission form.

Please respond directly to this email and provide any known details concerning your material's license terms and permissions required for reuse, even if you have not yet obtained copyright permissions or are unsure of your material's copyright compatibility. Once you have responded and addressed all other outstanding technical requirements, you may resubmit your manuscript within Editorial Manager.

Potential Copyright Issues:

i) Figure 1. Please confirm whether you drew the images / clip-art within the figure panels by hand. If you did not draw the images, please provide (a) a link to the source of the images or icons and their license / terms of use; or (b) written permission from the copyright holder to publish the images or icons under our CC BY 4.0 license. Alternatively, you may replace the images with open source alternatives. See these open source resources you may use to replace images / clip-art:

- https://commons.wikimedia.org

- https://openclipart.org/.

5) We note that your Data Availability Statement is currently as follows: "All data analysed or generated during this study are included in this article and its additional material". Please confirm at this time whether or not your submission contains all raw data required to replicate the results of your study. Authors must share the “minimal data set” for their submission. PLOS defines the minimal data set to consist of the data required to replicate all study findings reported in the article, as well as related metadata and methods (https://journals.plos.org/plosone/s/data-availability#loc-minimal-data-set-definition).

For example, authors should submit the following data:

1) The values behind the means, standard deviations and other measures reported;

2) The values used to build graphs;

3) The points extracted from images for analysis..

Authors do not need to submit their entire data set if only a portion of the data was used in the reported study.

If your submission does not contain these data, please either upload them as Supporting Information files or deposit them to a stable, public repository and provide us with the relevant URLs, DOIs, or accession numbers. For a list of recommended repositories, please see https://journals.plos.org/plosone/s/recommended-repositories.

If there are ethical or legal restrictions on sharing a de-identified data set, please explain them in detail (e.g., data contain potentially sensitive information, data are owned by a third-party organization, etc.) and who has imposed them (e.g., an ethics committee). Please also provide contact information for a data access committee, ethics committee, or other institutional body to which data requests may be sent. If data are owned by a third party, please indicate how others may request data access.

6) Please amend your detailed Financial Disclosure statement. This is published with the article. It must therefore be completed in full sentences and contain the exact wording you wish to be published.

1) State the initials, alongside each funding source, of each author to receive each grant. For example: "This work was supported by the National Institutes of Health (####### to AM; ###### to CJ) and the National Science Foundation (###### to AM)."

2) State what role the funders took in the study. If the funders had no role in your study, please state: "The funders had no role in study design, data collection and analysis, decision to publish, or preparation of the manuscript."

3) If any authors received a salary from any of your funders, please state which authors and which funders..

If you did not receive any funding for this study, please simply state: u201cThe authors received no specific funding for this work.u201d

7)  Please ensure that the funders and grant numbers match between the Financial Disclosure field and the Funding Information tab in your submission form. Note that the funders must be provided in the same order in both places as well.

**Reviewers' Comments:**

Reviewer's Responses to Questions

**Key Review Criteria Required for Acceptance?**

As you describe the new analyses required for acceptance, please consider the following:

**Methods**

-Are the objectives of the study clearly articulated with a clear testable hypothesis stated?

-Is the study design appropriate to address the stated objectives?

-Is the population clearly described and appropriate for the hypothesis being tested?

-Is the sample size sufficient to ensure adequate power to address the hypothesis being tested?

-Were correct statistical analysis used to support conclusions?

-Are there concerns about ethical or regulatory requirements being met?

Reviewer #1: See comments

Reviewer #2: (No Response)

Reviewer #3: The manuscript acknowledges that as a scoping review, it did not assess the risk of bias or methodological quality of the included studies. This is a major limitation for a review aiming to summarize and compare heterogeneous economic outcomes: without quality assessment, it is impossible to weigh how much to rely on each estimate. An assessment should be added and used to stratify or nuance the conclusions.

The lack of a registered protocol (e.g., in PROSPERO) reduces transparency and opens the door to selection and reporting bias. I recommend registering the protocol or justify the decision to the editor.

Limits of the bibliographic review. Why do you not have search in Web of science? Why not including non-english articles? The manuscript acknowledges incomplete capture of grey literature and reports contact with the World Mosquito Program and the use of Google Scholar. However, details are lacking about this opportunistic research. Please complete. Were institutional repositories, NGO reports, theses, ministry reports, or preprints searched? I suggest expanding the grey literature search and documenting the results.

**********

**Results**

-Does the analysis presented match the analysis plan?

-Are the results clearly and completely presented?

-Are the figures (Tables, Images) of sufficient quality for clarity?

Reviewer #1: See comments

Reviewer #2: (No Response)

Reviewer #3: The current review lists the results but does not provide a structured critical analysis explaining how differences in: how diseases were measured, disability weights used, whether vector control costs were included, the timeframes studied, and the assumptions about the intervention's effectiveness and duration affect the direction and size of the cost-effectiveness ratios.

In addition to descriptive tables, numerical analysis to answer relevant question. It would add depth and robustness to perform more complete analyses and figures that could be derived from the data extracted from the papers.

**********

**Conclusions**

-Are the conclusions supported by the data presented?

-Are the limitations of analysis clearly described?

-Do the authors discuss how these data can be helpful to advance our understanding of the topic under study?

-Is public health relevance addressed?

Reviewer #1: See comments

Reviewer #2: (No Response)

Reviewer #3: Lack of discussion on equity and operational feasibility: Cost-effectiveness alone is insufficient — governance, logistics, quality of the implementation, durability, program sustainability, community acceptance, regulatory issues, and equity considerations (e.g., urban vs. rural disparities or low vs high income areas) are not examined in depth.

I would also encourage the authors to address the broader issue of accessibility and democratization of the Wolbachia wMel technique. At present, implementation and large-scale deployment are primarily coordinated by a single established private organization, being accesible only for the governments or communities that can be pay this program. Therefore, it may limit transparency, competition, and the ability of countries to independently adopt or adapt the technology. For long-term sustainability and equitable global access, discussing this dimension is relevant. The potential conflicts of interest of reviewers might be more clearly stated, as in this meta-analysis, at least on of the authors receive funding for WMP, please be as transparente as possible.

**********

**Editorial and Data Presentation Modifications?**

Use this section for editorial suggestions as well as relatively minor modifications of existing data that would enhance clarity. If the only modifications needed are minor and/or editorial, you may wish to recommend “Minor Revision” or “Accept”.

Reviewer #1: Specific comments

P6, para 1 Suggest changing “These rely primarily on vector control” to “These rely primarily on vector control and vaccination.” Note: this revision promotes consistency with the finding in results (paragraph 2) that examines the combination of Wolbachia and vector control.

P6, para 1 After “less likely to transmit infection…” add comma there and after closing parenthesis

P10, Results, last paragraph. The authors may wish to change the phrase “above the effective threshold” to “above the economic threshold.”

P20 ref 13. For this and other references not in journals, provide further details (e.g. specifics and links of conference presentation and/or the abstract)

Supplementary materials, Table 1, see general comments.

Reviewer #2: Minor comments:

In the introduction – the last sentence in the second paragraph ‘A key difference from an operational and cost perspective is that the suppression-based strategy requires ongoing releases, whereas the replacement-based strategy does not’ should go in the methods, where the stratification of the different perspectives employed is described.

In the methods- I was a little bit confused as to why authors state that ‘No date or language restrictions were applied to the searches’ but then non-english publications were excluded at the extraction phase, particularly considering that trials have been implemented in Latin American countries.

Clarify in the inclusion/exclusion criteria that pre-prints are being included in the analysis

Define early on what is meant by smaller scale and make this categorization explicit in Table 2. Currently it’s not easy to determine how many studies were based on small areas, and how many on larger areas, which is critical to put the author’s conclusion (regarding cost-effectiveness in large urban settings) in context for the readers.

Check the typos: ‘high burden cites’ instead of cities throughout Table 1-3.

In first line of conclusion, sentence missing a verb: ‘when they appropriately targeted’.

For a better alignment with the journal scope: In the Discussion section, add a short subsection explicitly on equity and implementation; according to manuscript findings on cost-effectiveness, who would stand to be left out from cost-effectiveness-based decisions (e.g. rural poor), and what that implies for comprehensive dengue control strategies in endemic countries.

Reviewer #3: (No Response)

**********

**Summary and General Comments**

Use this section to provide overall comments, discuss strengths/weaknesses of the study, novelty, significance, general execution and scholarship. You may also include additional comments for the author, including concerns about dual publication, research ethics, or publication ethics. If requesting major revision, please articulate the new experiments that are needed.

Reviewer #1: Dengue scoping review – General comments

In a spirit of transparency, this reviewer is willing to provide his identify to the authors: Donald Shepard.

This reviewer commends the authors for incorporating updated information where available. For example, the last sentence of the section “Effectiveness of the intervention” noted that Wolbachia had remained established in Australia for 15 years as of the time of the authors’ submission.

This reviewer also commends the inclusion of the burden of persistent dengue in economic evaluations. They note that some economic studies have included that component and appropriately recommend further research to improve understanding of the underlying variability.

As established researchers, the authors are undoubtedly aware of the varied phrasing that different authors use in reporting their findings. They may wish to reflect this understanding more fully in classifying which findings are “not reported.” For example, in the authors’ Table 3, the authors indicate that the ICER was not reported for reference 12 (Shepard et al). However, Shepard et al. report the net cost for their 11 target cities as negative $134,139.468, i.e., a negative number. In this case, an ICER cannot be calculated, Shepard et al similarly note Wolbachia is cost saving in locations where the incidence is at least 500 symptomatic cases per 100,000 population per year. The “National” (i.e. all cities) estimate is substantially above this threshold.

The authors’ entry in Table 3 for Shepard et al (ref 12 in manuscript and ref 16 in supplementary material) give a benefit-cost ratio of 4.68. Shepard et al. also report aggregate costs, so that aggregate benefits in millions of dollars can be calculated from the information provided. The authors may wish to reconsider the entry “not reported.”

Similarly, while noting the cutoff dates for their review authors of scoping reviews sometimes incorporate updates or supplemental information. For example, the final version related to reference 12 was subsequently published as follows:

Shepard DS, Lee SR, Halasa-Rappel YA, Rincon Perez CW, Harker Roa A. Economic evaluation of Wolbachia deployment in Colombia: A modeling study. PLoS One 2025; 20(4): e0307045. https://doi.org/10.1371/journal.pone.0307045. Supporting information: https://doi.org/10.1371/journal.pone.0307045.s001 through s006

The manuscript uses the word “burden” 42 times. The authors’ text first uses the term “baseline burden” on p 6, where they note that they abstracted the “baseline burden of symptomatic cases.” As an area-based vector control technology, Wolbachia depends two measures of burden: dengue incidence per 100,000 population and population density (persons per kilometer). Shepard et al (ref 12, Fig 5B of published version) show how the combination of these two measures lead to a high “burden” and the most favorable benefit-cost ratios (the highest, 8.85 in the city of Bucaramanga, with the highest population density). This reviewer urges the authors to consider whether this two-dimensional definition of burden deserves highlighting for policy recommendations.

In comparing the replacement versus suppression Wolbachia strategies (p11), this reviewer encourages the authors to offer more policy insights about the results to date on this important question. For example, their discussion could suggest how the results on the suppression strategy from Singapore might be adapted to low- and middle-income countries to be compared with the replacement strategies. Similarly, in discussing the interplay between vaccination and Wolbachia, the cost structure of the two preventive interventions already generates important insights. Wolbachia costs depend primary on the square kilometers covered, whereas vaccination costs depend on the number of persons vaccinated. These patterns suggest that Wolbachia may be preferable in dense urban areas, whereas vaccination may be especially cost effective in areas of high dengue incidence but lower population density. The authors’ conclusions that “Further studies are needed…” (p11) and “There is a need to investigate…” (p 18) are always true and apply to every topic. This paper should maximize its contribution to policy as a careful and comprehensive review.

The variability in the effectiveness of Wolbachia is an important consideration, as the authors note on p15. However, a more balanced discussion would be helpful. The authors may also want to discuss experience from observational studies around Medellin, Colombia, summarized in the publication above by Shepard et al (2025). By avoiding some of the movement factors noted from Yogyakarta, Wolbachia proved more effective in Colombia.

Reviewer #2: This manuscript presents a scoping review of economic evaluations on the cost-effectiveness of Wolbachia-based interventions, noting that the results indicate that this control method is cost-effective in large urban settings. This is a welcome discussion, especially considering the urgency of addressing rising cases of dengue and need for evidence-based policy regarding effective mosquito control methods. My main concern is the use strong language indicating a high degree of support for findings that are presented in a mostly qualitative and narrative manner, and based on a review of heterogeneous and small number of studies.

For example, the authors use the word ‘consensus’ in the abstract, author summary and discussion to indicate that ‘there is consensus that Wolbachia-based replacement interventions can be cost-effective for dengue control when targeted to high-burden settings’, but the evidence base is small, with heterogeneity in assumptions of baseline burdens as the authors themselves recognise. I would suggest rephrasing as ‘the available economic evaluations consistently suggest’, even more so considering that, to determine cost-effectiveness of the intervention, authors seem to be mostly comparing against a single numerical rule (0.5 threshold), for which there is some criticism, particularly regarding the need for such thresholds to reflect a country’s budget and context. However, this is not discussed as part of the assumptions made by the study, nor in areas that need further research section. I strongly recommend that authors justify this choice in more detail, with the appropriate interpretation caveats, as these studies have been conducted in countries with very different settings and especially because this manuscript could be of interest for decision-makers considering this control strategy.

Another major comment pertains to mixing cost years and currencies without standardisation. Costs in Table 2 are reported in US$ but without specifying price years (this is sometimes specified in the manuscript’s text, but not for every estimate presented). Considering the wording of some results, such as ‘The assumed base case total cost per person covered within the evaluations of the replacement-based strategy varied between US$2.47-44.5 per person..’, I couldn’t determine if these values were standardised to a common price year, or underwent inflation adjustment. Ideally, authors would convert all costs to a common year and state this in methods.

The mostly narrative style of the manuscript and the table-format presentation means the reader must mentally integrate long textual descriptions and scan through large tables for each setting’s population size, cost per person and other important information to draw our own conclusions. I strongly suggest including some basic data visualization to improve clarity and support the manuscript conclusions. Perhaps a figure plotting cost per person protected vs. population size.

Reviewer #3: This is a relevant and original article as there does not exist a scoping revision of economic evaluations of Wolbachia-based interventions for dengue control, being for interest in the field of NTDS but also for researchers or practitioners outside the field. The manuscript requires significant revisions to improve its methodological rigor and analytical depth. This is a major limitation for a review comparing economic outcomes and undermines the reliability of its conclusions. To strengthen the work, the authors must (1) formally assess the quality of included studies using an appropriate tool and use this assessment to inform the interpretation of results. (2) The lack of a registered protocol should be explicitly justified in the text, and the study protocol should be provided as supplementary material. (3) The search strategy must be expanded to include a more systematic and documented coverage of grey literature (e.g., institutional reports, theses), detailing precisely what was searched and what was found. (4) The synthesis should move beyond listing results; where data permit, results should be standardized into comparable metrics like ICERs. Where direct comparison is not possible, the limitations must be clearly stated to avoid unqualified qualitative comparisons. Potential statistical graphs or analysis can be made from the data and it is worth to include them, and also to made accesible to the data in a repository. Finally, (5) the discussion must be expanded to include a critical analysis of how key drivers of heterogeneity—such as the assumed duration of effect and variation in effectiveness estimates—influence the conclusions, using concrete examples from the reviewed studies.

**********

PLOS authors have the option to publish the peer review history of their article (what does this mean?). If published, this will include your full peer review and any attached files.

If you choose “no”, your identity will remain anonymous but your review may still be made public.

**Do you want your identity to be public for this peer review?** For information about this choice, including consent withdrawal, please see our Privacy Policy.

Reviewer #1: **Yes:** Donald S Shepard

Reviewer #2: No

Reviewer #3: No

**Figure resubmission:**

While revising your submission, we strongly recommend that you use PLOS’s NAAS tool (https://ngplosjournals.pagemajik.ai/artanalysis) to test your figure files. NAAS can convert your figure files to the TIFF file type and meet basic requirements (such as print size, resolution), or provide you with a report on issues that do not meet our requirements and that NAAS cannot fix.

After uploading your figures to PLOS’s NAAS tool - https://ngplosjournals.pagemajik.ai/artanalysis, NAAS will process the files provided and display the results in the "Uploaded Files" section of the page as the processing is complete. If the uploaded figures meet our requirements (or NAAS is able to fix the files to meet our requirements), the figure will be marked as "fixed" above. If NAAS is unable to fix the files, a red "failed" label will appear above. When NAAS has confirmed that the figure files meet our requirements, please download the file via the download option, and include these NAAS processed figure files when submitting your revised manuscript.

**Reproducibility:**

To enhance the reproducibility of your results, we recommend that authors of applicable studies deposit laboratory protocols in protocols.io, where a protocol can be assigned its own identifier (DOI) such that it can be cited independently in the future. Additionally, PLOS ONE offers an option to publish peer-reviewed clinical study protocols. Read more information on sharing protocols at https://plos.org/protocols?utm_medium=editorial-email&utm_source=authorletters&utm_campaign=protocols

# Author comment: Author response to Decision Letter 1

**DOI:** 10.1371/journal.pntd.0014395.r002

20 Feb 2026

Attachment Submitted filename: Wol health econ Comments.docx

# Peer review: Decision Letter 1

**DOI:** 10.1371/journal.pntd.0014395.r003

**Authors:** Nigel Beebe, Gordana Rasic

22 Apr 2026

PNTD-D-25-01795R1

The cost-effectiveness of Wolbachia-based biocontrol interventions for dengue: A scoping review of the available evidence

PLOS Neglected Tropical Diseases

Dear Dr. Turner,

Thank you for submitting your manuscript to PLOS Neglected Tropical Diseases. After careful consideration, we feel that it has merit but does not fully meet PLOS Neglected Tropical Diseases's publication criteria as it currently stands. Therefore, we invite you to submit a revised version of the manuscript that addresses the points raised during the review process.

Please submit your revised manuscript by May 22 2026 11:59PM. If you will need more time than this to complete your revisions, please reply to this message or contact the journal office at plosntds@plos.org. When you're ready to submit your revision, log on to https://www.editorialmanager.com/pntd/ and select the 'Submissions Needing Revision' folder to locate your manuscript file.

Please include the following items when submitting your revised manuscript:

* A letter that responds to each point raised by the editor and reviewer(s). You should upload this letter as a separate file labeled 'Response to Reviewers'. This file does not need to include responses to any formatting updates and technical items listed in the 'Journal Requirements' section below.

* A marked-up copy of your manuscript that highlights changes made to the original version. You should upload this as a separate file labeled 'Revised Manuscript with Track Changes'.

* An unmarked version of your revised paper without tracked changes. You should upload this as a separate file labeled 'Manuscript'.

If you would like to make changes to your financial disclosure, competing interests statement, or data availability statement, please make these updates within the submission form at the time of resubmission. Guidelines for resubmitting your figure files are available below the reviewer comments at the end of this letter.

We look forward to receiving your revised manuscript.

Kind regards,

Gordana Rasic, Ph.D.

Academic Editor

PLOS Neglected Tropical Diseases

Nigel Beebe

Section Editor

PLOS Neglected Tropical Diseases

Shaden Kamhawi

co-Editor-in-Chief

PLOS Neglected Tropical Diseases

orcid.org/0000-0003-4304-636XX

Paul Brindley

co-Editor-in-Chief

PLOS Neglected Tropical Diseases

orcid.org/0000-0003-1765-0002

**Additional Editor Comments:**

The manuscript is well-executed, clearly written, and makes a valuable and policy-relevant contribution to the field. The reviewers are broadly supportive, and the requested at this stage revisions are Minor and primarily focused on clarity, consistency, and strengthening the alignment between results and interpretation. Please refer to these priority revisions for final acceptance:

1) Strengthen the abstract and key messaging:

• Make the main findings more explicit and informative (particularly cost-saving implications), and replace vague or generic concluding statements with specific takeaways.

• Align conclusions with the evidence presented.

• Ensure the discussion accurately reflects the strength of the findings, particularly regarding the consistent cost-effectiveness of replacement strategies, and avoid unnecessary calls for further research where evidence is already clear.

2) Enhance clarity, transparency, and consistency:

• Address minor issues in wording precision, referencing (including use of final published sources), and table reporting (e.g., clearer terminology and inclusion of simple derived metrics where appropriate).

**Journal Requirements:**

If the reviewer comments include a recommendation to cite specific previously published works, please review and evaluate these publications to determine whether they are relevant and should be cited. There is no requirement to cite these works unless the editor has indicated otherwise.

**Reviewers' comments:**

Reviewer's Responses to Questions

**Key Review Criteria Required for Acceptance?**

As you describe the new analyses required for acceptance, please consider the following:

**Methods**

-Are the objectives of the study clearly articulated with a clear testable hypothesis stated?

-Is the study design appropriate to address the stated objectives?

-Is the population clearly described and appropriate for the hypothesis being tested?

-Is the sample size sufficient to ensure adequate power to address the hypothesis being tested?

-Were correct statistical analysis used to support conclusions?

-Are there concerns about ethical or regulatory requirements being met?

Reviewer #1: Yes. The methods of this scoping review are appropriate. This reviewer commends the many improvements reflected in this revision.

Reviewer #3: Yes

**********

**Results**

-Does the analysis presented match the analysis plan?

-Are the results clearly and completely presented?

-Are the figures (Tables, Images) of sufficient quality for clarity?

Reviewer #1: Results only partially match the analysis plan. The very different results between the replacement and suppression strategies needs further highlighting. As authors who seek to inform policy, they should make maximum use of information which is indirectly reported to estimate the long term cost-effectiveness of the suppression strategy and compare it to the replacement strategy.

Reviewer #3: Yes

**********

**Conclusions**

-Are the conclusions supported by the data presented?

-Are the limitations of analysis clearly described?

-Do the authors discuss how these data can be helpful to advance our understanding of the topic under study?

-Is public health relevance addressed?

Reviewer #1: The revision is improved over the previous version. Nevertheless, the comment from results applies to the conclusions and is repeated here:

The very different results between the replacement and suppression strategies needs further highlighting. As authors who seek to inform policy, they should make maximum use of information which is indirectly reported to estimate the long term cost-effectiveness of the suppression strategy and compare it to the replacement strategy.

Reviewer #3: Yes

**********

**Editorial and Data Presentation Modifications?**

Use this section for editorial suggestions as well as relatively minor modifications of existing data that would enhance clarity. If the only modifications needed are minor and/or editorial, you may wish to recommend “Minor Revision” or “Accept”.

Reviewer #1: Note: This reviewer used the page numbers in the electronic pdf version, which includes four background pages before the revised draft manuscript. Thus, PDF p 5 is the title page (p 1 as labeled by the authors).

Pdf P 1 The following phase in the abstract is currently in parentheses: (with the intervention likely to generate cost savings from a societal perspective). Given the importance of this finding, it should not be enclosed in parentheses, but instead separated by comma.

Pdf P 1 (abstract) and P 7 (authors’ summary). As the authors know, most readers seek informative abstracts. Most of the abstract meets this expectation, but the last sentence, which follows, falls short: “We outline recommendations for future economic evaluations in this area.” This reviewer recommends that that sentence, be deleted and the previous sentence be extended by beginning with the word “including” and inserting the most important recommendation.

Pdf P 2, Instead of “to date,” this reviewer suggests inserting the date of the authors’ cutoff date (29 April 2024).

Pdf p 10. To avoid ambiguity, this reviewer suggests changing “Greater than 200,000…” to “At least 200,000…”

PDF p 10. A more recent publication strengthens the authors’ choice of a threshold of 0.5 GDP/capita (currently based on refs 15 and 16). This reviewer suggests that the authors include a statement along the following lines: “An empirical analysis of historical spending across 174 countries found that that 51% implicitly used thresholds of cost per QALY of 0.5 times their per capita GDP (Pichon-Riviere et al., 2023).”

Pichon-Riviere A, Drummond M, Palacios A, Garcia-Marti S, Augustovski F. Determining the efficiency path to universal health coverage: cost-effectiveness thresholds for 174 countries based on growth in life expectancy and health expenditures. Lancet Glob Health. 2023;11(6):e833-e42. doi:10.1016/S2214-109X(23)00162-6.

Pdf p 11. This reviewer agrees with the authors’ decision to include subsequent published versions of papers that had not been peer reviewed at the time of the authors’ search. As the authors indicated that they used the published version for their search, their citations in the text and tables should be broadened to include the published version in all places. For example, pdf p 11 refers only to the preprint version from Colombia (ref 25) but not the published version (ref 47).

Pdf p 16. This reviewer agrees only in part with the authors’ conclusion that Wolbachia studies have not generally addressed distributional elements. However, it would be helpful for the authors to add that Wolbachia is an area-based intervention that does not require any action from a resident to benefit. Therefore, it is likely to benefit all residents in the target area similarly. Accordingly, the authors could draw on an interview study across eight endemic countries in both Asia and the Americas (Suaya JA et al. Am. J. Trop. Med. Hyg., 80(5), 2009, pp. 846–855). In that study, 83% of the participants were urban, similar to the potential populations served by Wolbachia. That study found that 29% of respondents had primary education or less, 44% secondary, and 27% vocational or higher. Thus, it is likely the equity considerations of Wolbachia would be broad, benefiting residents across all socio-economic levels.

Pdf p 17. The authors’ final result states that further studies are needed concerning the relative cost-effectiveness of replacement versus suppression. That statement is not consistent with the results of their scoping review, which finds that replacement is consistently cost-effective, and sometimes cost-saving. Furthermore, the authors should comment on Singapore’s previous suppression strategy through the country’s vigorous through intensive efforts to monitor and eliminate breeding sites through inspections and penalties. These policies had the perverse effect of lowering population immunity, contributing to subsequent large outbreaks. Furthermore, the authors noted that most suppression strategies assume a 10-year period of effectiveness. This, if program costs are annualized, the suppression approach is about ten times more costly than the replacement approach. If the calculations recommended below for pdf pp 38-41 are completed, I expect that the suppression approach will not be cost saving even though Singapore is a dense city. This paper would best serve the goal of informing policy through reasonable projections, with caveats, instead of the cliché of calling for more research.

Pdf p 18. The authors wrote: The cost-effectiveness ratios from a societal perspective were often negative….” To be precise, the authors should consider inserting “of the replacement strategy….”

PDF P 27. Following recommended style, the authors have generally italicized the word Wolbachia. They should check their manuscript and improve consistency for places like the beginning of the second full paragraph on this page, where they did not italicize “Wolbachia-based.”

Pdf p 38-41. In Tables 2, the term “Not explicitly reported” is misleading. It would be more accurate to state “Indirectly reported.” By indicating that the program is national, the authors provide the data for deriving the underlying population, the cost per person, and benefit cost ratios. With the paper’s goal of informing the field, these simple calculations should be done to fill in the missing items, with explanations provided in footnotes or supplements.

Reviewer #3: Accept

**********

**Summary and General Comments**

Use this section to provide overall comments, discuss strengths/weaknesses of the study, novelty, significance, general execution and scholarship. You may also include additional comments for the author, including concerns about dual publication, research ethics, or publication ethics. If requesting major revision, please articulate the new experiments that are needed.

Reviewer #1: Comments to authors (copied from above):

Note: This reviewer used the page numbers in the electronic pdf version, which includes four background pages before the revised draft manuscript. Thus, PDF p 5 is the title page (p 1 as labeled by the authors).

Pdf P 1 The following phase in the abstract is currently in parentheses: (with the intervention likely to generate cost savings from a societal perspective). Given the importance of this finding, it should not be enclosed in parentheses, but instead separated by comma.

Pdf P 1 (abstract) and P 7 (authors’ summary). As the authors know, most readers seek informative abstracts. Most of the abstract meets this expectation, but the last sentence, which follows, falls short: “We outline recommendations for future economic evaluations in this area.” This reviewer recommends that that sentence, be deleted and the previous sentence be extended by beginning with the word “including” and inserting the most important recommendation.

Pdf P 2, Instead of “to date,” this reviewer suggests inserting the date of the authors’ cutoff date (29 April 2024).

Pdf p 10. To avoid ambiguity, this reviewer suggests changing “Greater than 200,000…” to “At least 200,000…”

PDF p 10. A more recent publication strengthens the authors’ choice of a threshold of 0.5 GDP/capita (currently based on refs 15 and 16). This reviewer suggests that the authors include a statement along the following lines: “An empirical analysis of historical spending across 174 countries found that that 51% implicitly used thresholds of cost per QALY of 0.5 times their per capita GDP (Pichon-Riviere et al., 2023).”

Pichon-Riviere A, Drummond M, Palacios A, Garcia-Marti S, Augustovski F. Determining the efficiency path to universal health coverage: cost-effectiveness thresholds for 174 countries based on growth in life expectancy and health expenditures. Lancet Glob Health. 2023;11(6):e833-e42. doi:10.1016/S2214-109X(23)00162-6.

Pdf p 11. This reviewer agrees with the authors’ decision to include subsequent published versions of papers that had not been peer reviewed at the time of the authors’ search. As the authors indicated that they used the published version for their search, their citations in the text and tables should be broadened to include the published version in all places. For example, pdf p 11 refers only to the preprint version from Colombia (ref 25) but not the published version (ref 47).

Pdf p 16. This reviewer agrees only in part with the authors’ conclusion that Wolbachia studies have not generally addressed distributional elements. However, it would be helpful for the authors to add that Wolbachia is an area-based intervention that does not require any action from a resident to benefit. Therefore, it is likely to benefit all residents in the target area similarly. Accordingly, the authors could draw on an interview study across eight endemic countries in both Asia and the Americas (Suaya JA et al. Am. J. Trop. Med. Hyg., 80(5), 2009, pp. 846–855). In that study, 83% of the participants were urban, similar to the potential populations served by Wolbachia. That study found that 29% of respondents had primary education or less, 44% secondary, and 27% vocational or higher. Thus, it is likely the equity considerations of Wolbachia would be broad, benefiting residents across all socio-economic levels.

Pdf p 17. The authors’ final result states that further studies are needed concerning the relative cost-effectiveness of replacement versus suppression. That statement is not consistent with the results of their scoping review, which finds that replacement is consistently cost-effective, and sometimes cost-saving. Furthermore, the authors should comment on Singapore’s previous suppression strategy through the country’s vigorous through intensive efforts to monitor and eliminate breeding sites through inspections and penalties. These policies had the perverse effect of lowering population immunity, contributing to subsequent large outbreaks. Furthermore, the authors noted that most suppression strategies assume a 10-year period of effectiveness. This, if program costs are annualized, the suppression approach is about ten times more costly than the replacement approach. If the calculations recommended below for pdf pp 38-41 are completed, I expect that the suppression approach will not be cost saving even though Singapore is a dense city. This paper would best serve the goal of informing policy through reasonable projections, with caveats, instead of the cliché of calling for more research.

Pdf p 18. The authors wrote: The cost-effectiveness ratios from a societal perspective were often negative….” To be precise, the authors should consider inserting “of the replacement strategy….”

PDF P 27. Following recommended style, the authors have generally italicized the word Wolbachia. They should check their manuscript and improve consistency for places like the beginning of the second full paragraph on this page, where they did not italicize “Wolbachia-based.”

Pdf p 38-41. In Tables 2, the term “Not explicitly reported” is misleading. It would be more accurate to state “Indirectly reported.” By indicating that the program is national, the authors provide the data for deriving the underlying population, the cost per person, and benefit cost ratios. With the paper’s goal of informing the field, these simple calculations should be done to fill in the missing items, with explanations provided in footnotes or supplements.

Reviewer #3: (No Response)

**********

PLOS authors have the option to publish the peer review history of their article (what does this mean?). If published, this will include your full peer review and any attached files.

If you choose “no”, your identity will remain anonymous but your review may still be made public.

**Do you want your identity to be public for this peer review?** For information about this choice, including consent withdrawal, please see our Privacy Policy.

Reviewer #1: **Yes:** Prof. Donald S. Shepard

Reviewer #3: No

**Figure resubmission:**

While revising your submission, we strongly recommend that you use PLOS’s NAAS tool (https://ngplosjournals.pagemajik.ai/artanalysis) to test your figure files. NAAS can convert your figure files to the TIFF file type and meet basic requirements (such as print size, resolution), or provide you with a report on issues that do not meet our requirements and that NAAS cannot fix.

After uploading your figures to PLOS’s NAAS tool - https://ngplosjournals.pagemajik.ai/artanalysis, NAAS will process the files provided and display the results in the "Uploaded Files" section of the page as the processing is complete. If the uploaded figures meet our requirements (or NAAS is able to fix the files to meet our requirements), the figure will be marked as "fixed" above. If NAAS is unable to fix the files, a red "failed" label will appear above. When NAAS has confirmed that the figure files meet our requirements, please download the file via the download option, and include these NAAS processed figure files when submitting your revised manuscript.

**Reproducibility:**

To enhance the reproducibility of your results, we recommend that authors of applicable studies deposit laboratory protocols in protocols.io, where a protocol can be assigned its own identifier (DOI) such that it can be cited independently in the future. Additionally, PLOS ONE offers an option to publish peer-reviewed clinical study protocols. Read more information on sharing protocols at https://plos.org/protocols?utm_medium=editorial-email&utm_source=authorletters&utm_campaign=protocols

# Author comment: Author response to Decision Letter 2

**DOI:** 10.1371/journal.pntd.0014395.r004

13 May 2026

Attachment Submitted filename: Wol health econ Comments round 2_hc.docx

# Editor report: Decision Letter 2

**DOI:** 10.1371/journal.pntd.0014395.r005

**Authors:** Nigel Beebe, Gordana Rasic

18 May 2026

Dear Dr. Turner,

We are pleased to inform you that your manuscript 'The cost-effectiveness of Wolbachia-based biocontrol interventions for dengue: A scoping review of the available evidence' has been provisionally accepted for publication in PLOS Neglected Tropical Diseases.

Before your manuscript can be formally accepted you will need to complete some formatting changes, which you will receive in a follow up email. A member of our team will be in touch with a set of requests.

Please note that your manuscript will not be scheduled for publication until you have made the required changes, so a swift response is appreciated.

IMPORTANT: The editorial review process is now complete. PLOS will only permit corrections to spelling, formatting or significant scientific errors from this point onwards. Requests for major changes, or any which affect the scientific understanding of your work, will cause delays to the publication date of your manuscript.

Should you, your institution's press office or the journal office choose to press release your paper, you will automatically be opted out of early publication. We ask that you notify us now if you or your institution is planning to press release the article. All press must be co-ordinated with PLOS.

Thank you again for supporting Open Access publishing; we are looking forward to publishing your work in PLOS Neglected Tropical Diseases.

Best regards,

Gordana Rasic, Ph.D.

Academic Editor

PLOS Neglected Tropical Diseases

Nigel Beebe

Section Editor

PLOS Neglected Tropical Diseases

Shaden Kamhawi

co-Editor-in-Chief

PLOS Neglected Tropical Diseases

orcid.org/0000-0003-4304-636XX

Paul Brindley

co-Editor-in-Chief

PLOS Neglected Tropical Diseases

orcid.org/0000-0003-1765-0002

***********************************************************

p.p1 {margin: 0.0px 0.0px 0.0px 0.0px; line-height: 16.0px; font: 14.0px Arial; color: #323333; -webkit-text-stroke: #323333}span.s1 {font-kerning: none

# Editor report: Acceptance letter

**DOI:** 10.1371/journal.pntd.0014395.r006

**Authors:** Nigel Beebe, Gordana Rasic

Dear Dr. Turner,

We are delighted to inform you that your manuscript, "The cost-effectiveness of Wolbachia-based biocontrol interventions for dengue: A scoping review of the available evidence," has been formally accepted for publication in PLOS Neglected Tropical Diseases.

We have now passed your article onto the PLOS Production Department who will complete the rest of the publication process. All authors will receive a confirmation email upon publication.

The corresponding author will soon be receiving a typeset proof for review, to ensure errors have not been introduced during production. Please review the PDF proof of your manuscript carefully, as this is the last chance to correct any scientific or type-setting errors. Please note that major changes, or those which affect the scientific understanding of the work, will likely cause delays to the publication date of your manuscript. Note: Proofs for Front Matter articles (Editorial, Viewpoint, Symposium, Review, etc...) are generated on a different schedule and may not be made available as quickly.

Soon after your final files are uploaded, the early version of your manuscript will be published online unless you opted out of this process. The date of the early version will be your article's publication date. The final article will be published to the same URL, and all versions of the paper will be accessible to readers.

For Research Articles, you will receive an invoice from PLOS for your publication fee after your manuscript has reached the completed accept phase. If you receive an email requesting payment before acceptance or for any other service, this may be a phishing scheme. Learn how to identify phishing emails and protect your accounts at https://explore.plos.org/phishing.

Thank you again for supporting open-access publishing; we are looking forward to publishing your work in PLOS Neglected Tropical Diseases.

Best regards,

Shaden Kamhawi

co-Editor-in-Chief

PLOS Neglected Tropical Diseases

Paul Brindley

co-Editor-in-Chief

PLOS Neglected Tropical Diseases
